# Integrated 3D static modelling to assess hydrocarbon potential of the fluvial-alluvial sandstones of Nukhul Formation, October oil field, Gulf of Suez, Egypt

**DOI:** 10.1038/s41598-026-42298-1

**Published:** 2026-03-30

**Authors:** Mostafa A. Khattab, Ahmed E. Radwan, Mohamed I. El-Anbaawy, Adel A. El-Tehiwy

**Affiliations:** 1 Geology department, Faculty of science, Cairo university , Giza , Egypt; 2 Faculty of Geography and Geology, Institute of Geological Sciences, Jagiellonian University, Kraków, Poland; 3 Geology Department, Faculty of Science, Cairo University, Giza, Egypt

**Keywords:** Planetary science, Solid Earth sciences

## Abstract

One of the biggest hydrocarbon accumulations in the Gulf of Suez, the huge October Oil Field, has a structurally complex syn-rift sequence with poorly limited reservoir distribution and quality. One of the most important target reservoirs is the Nukhul Formation. However, the field development is challenged by facies architecture, porosity, shale distribution, and fault geometries that complicate replication of the Nukhul reservoir’s reported heterogeneity. These difficulties are crucial for enhancing and improving field development, particularly from the Nukhul reservoir, which was depleted and had a very low oil rate. It was thought that this type of reservoir had disappeared, leaving little opportunity for development. This study aims to manage and increase oil production from the Nukhul reservoir by updating structural interpretation, which reveals revised fault geometry and compartment configurations, providing better restrictions on trap integrity and reservoir continuity. Historical datasets collected by multiple operators (1980–2020), combined with recently reprocessed seismic interpretations, were integrated to reconstruct stratigraphic geometries more accurately than previously achievable. It was crucial to update and modify the structural model to preserve favourable reservoir quality and extension across different locations, forming the basis for improved facies and static modelling. The Nukhul Formation was divided into four zones (K1–K4) based on detailed correlation integrated with petrological descriptions and dynamic data. This division highlights the dominance of low-porosity limestone–shale units in K1–K2, discontinuous fluvial sandstone bodies in K3, and thick, laterally linked channelized sandstones in K4. Model results identify undrilled reservoir extensions, attic accumulations, and high-quality sandstone corridors. These results provide actionable targets for near-field exploration and infill drilling, significantly reducing uncertainty in reservoir extent and quality, improving dynamic behaviour prediction, and supporting volumetric calculations, flow-unit delineation, and uncertainty quantification. The findings demonstrate that the Nukhul reservoir still retains production potential and can contribute to field redevelopment. The approach used here offers a stable and portable framework for characterizing heterogeneous syn-rift reservoirs in structurally dynamic basin-margin environments and promotes optimal field redevelopment plans.

## Introduction

The Nukhul Formation is one of the most significant target reservoirs in Egypt’s Gulf of Suez, particularly in the October oil field (the study area). For a variety of reasons, the Nukhul reservoir turned into a low-rate producer of hydrocarbons. This study addresses the critical challenge of declining production caused by inadequate characterization of reservoir heterogeneity and structural complexity. In order to give a clear and workable field depletion strategy, this study intends to manage and grow the oil output from the Nukhul reservoir, develop strategies to improve its quality, and assess its findings. If the current structural model is not updated and a realistic facies model is not established, a significant amount of hydrocarbons that could be drained using alternative development concepts will be neglected, leading to suboptimal field performance and negative consequences for the oil industry. Additionally, this approach may open the door for similar work in neighbouring fields.

Static three-dimensional (3D) reservoir modelling workflows are fundamental for distributing facies and porosity and for quantifying lateral reservoir connectivity and porosity enhancement trends^1–5^. These spatial characterizations are essential inputs for reservoir architecture and heterogeneity, volumetric calculations, uncertainty quantification, and facies distribution risk assessment in petroleum systems. Earlier investigations provided basic insights but were constrained by the well control and seismic resolution available at the time, including studies conducted by GUPCO and BP for the Miocene section in 2015. Geoscientists have applied Monte Carlo and geostatistical approaches to generate multiple equiprobable realizations and probabilistic reserve estimates, enabling risk-informed decision-making for exploration and field development^4,6,7^. Recently^8^, integrated 3D structural modelling and seismic interpretation to optimize hydrocarbon development in the Early Miocene Nukhul Formation; however, detailed facies modelling and reservoir-scale petrophysical heterogeneity were not investigated. Despite these advances, 3D modelling accuracy and uncertainty remain major limitations, requiring iterative updates with new seismic and well data to improve model reliability^4^.

Building on recent seismic reprocessing and newly drilled wells, the present research focuses on updated 3D static modelling of the Lower Miocene Nukhul Formation to enhance hydrocarbon reservoir assessment and simulation. By coupling multidisciplinary data and contemporary geostatistical tools, the updated static model is designed to (a) reduce uncertainty in reserve estimates, (b) delineate flow units and structural compartments for depletion planning, and (c) identify prospective targets for future appraisal and development. Net-to-gross, water-saturation, and porosity maps derived from integrated petrophysical, geological, geophysical, and reservoir engineering analyses constitute primary inputs to constrain reservoir architecture and heterogeneity^1,9–16^. By adding detailed facies modelling and petrophysical zonation, this study improves the workflow compared to^8^, which mainly concentrated on structural and seismic interpretations. This resolves reservoir-scale heterogeneity and provides a more comprehensive static model that enhances redevelopment planning in mature syn-rift reservoirs.

## Geological setting

**Fig. 1:**
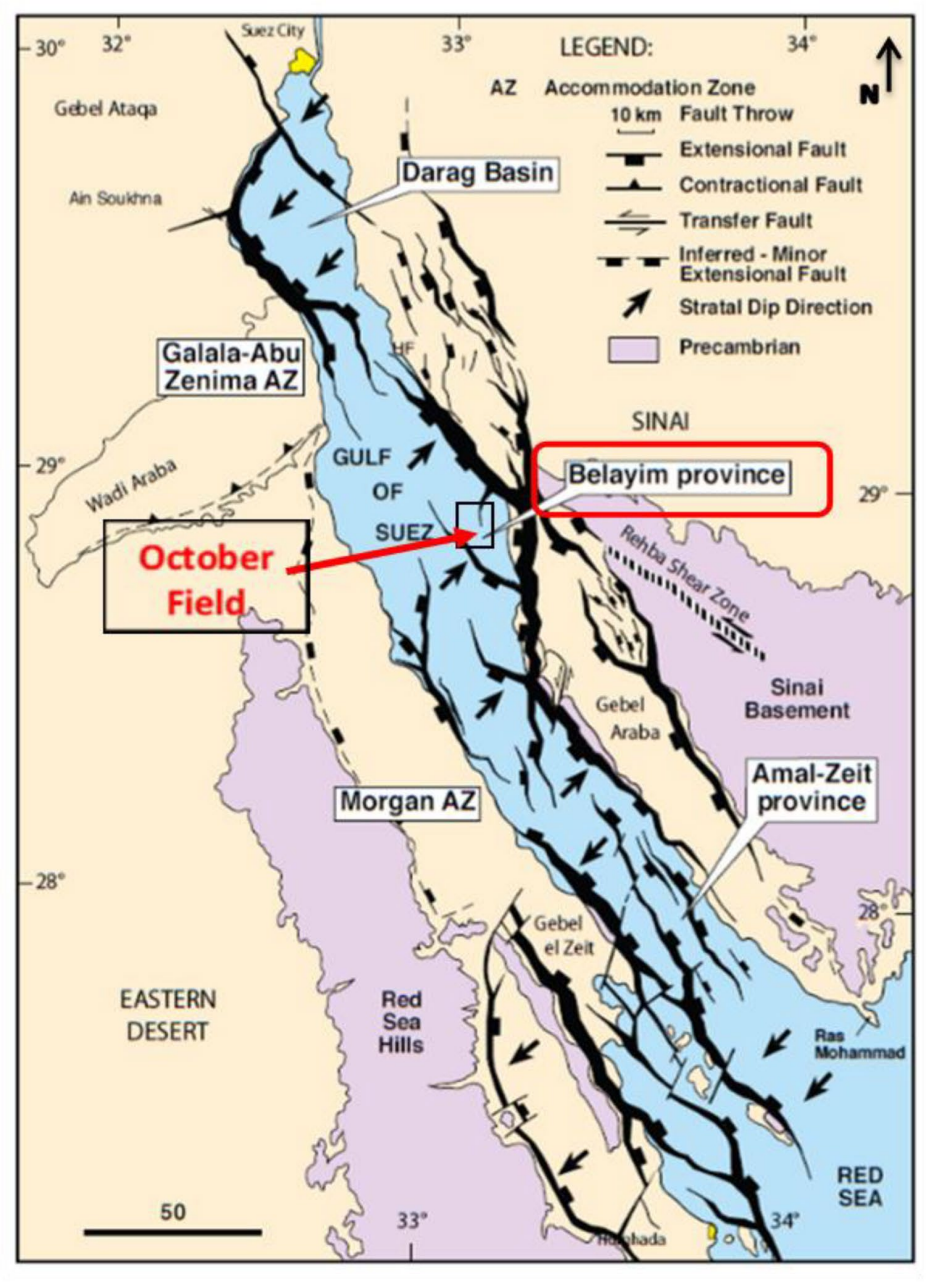
The three main provinces around the Gulf of Suez are the Darag basin in the north, the Belayim basin in the middle, and the Amal-Zeit basin in the south. The October oil field, the subject of this study, is located in the Belayim province basin.^25^.

**Fig. 2:**
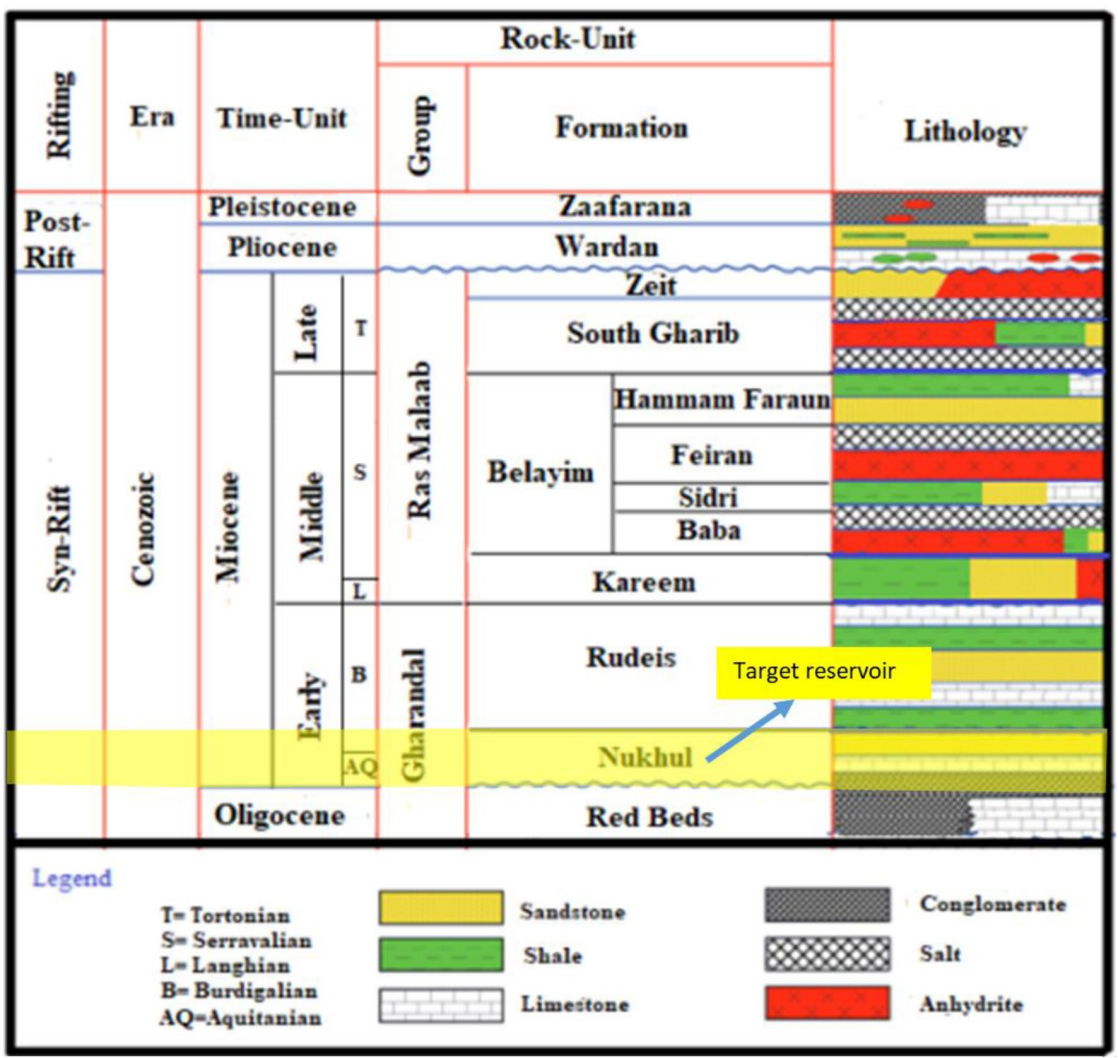
The lithostratigraphic column, which represents the entire section for the Miocene that showing the Nukhul target reservoir (target of study) within the Gulf of Suez, Egypt.

The October Oil Field is located in Egypt’s Gulf of Suez, between latitudes 28° 45′ and 28° 55′ N and longitudes 33° 00′ and 33° 10′ E **(**Fig. 1**)**. It is regarded as the third-largest oil field in the Gulf of Suez region after the Belayim Marine and Morgan oil fields. Since its discovery in 1977, Egypt’s hydrocarbon production has relied heavily on the October Oil Field, whose structural traps are mostly associated with tilted fault blocks^17^. This major oil field spans an area of thirty kilometers and consists of nine distinct fault blocks as well as a few smaller stratigraphic traps. Multiple reservoir intervals drive the field’s prolific character, with the Miocene sequences—especially Asl and Kareem Formations—contributing heavily to its cumulative output. The geologic succession in the Gulf of Suez spans from the Precambrian to the Holocene. The October Oil Field’s stratigraphic column shows that the main reservoirs, from bottom to top, are the Nubia sandstone, Nezazzat group, Nukhul Formation, Upper Rudeis Formation, and Belayim Formation, as shown in **(**Fig. 2**)**. The area of study is the OCT-D block, which is located in the middle of the October trend. The October Oil Field’s complex faulting system characterizes the main hydrocarbon accumulations in addition to providing compartmentalization, necessitating thorough structural modelling for efficient development ^18^. The sedimentary systems, structural configuration, and reservoir characterization of these fault blocks have been further clarified by recent research, emphasizing their influence on depositional environments, reservoir fluid flow and reserve distribution^1,19–24^. Production was established from the OCT-D1 exploration well from the Nukhul sandstone reservoir, which has a gross oil column of approximately 110 ft and produces 32° API oil.

**Fig. 3:**
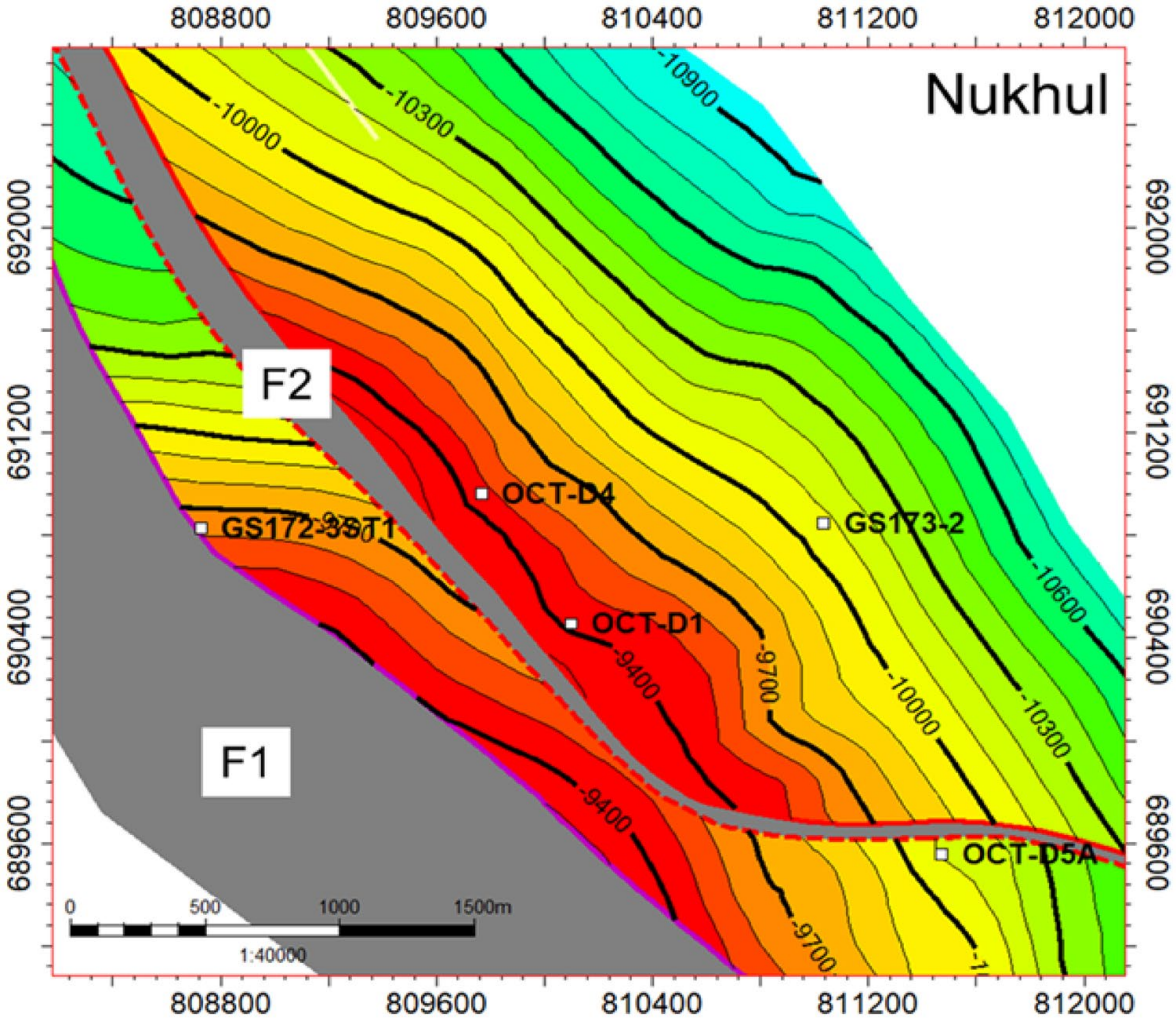
Structure contour map on Nukhul level, showing the selected five wells for the current study (from the current work interpretation).

Several distinct reservoir accumulations are present in the study area. The sandstone of the Nukhul Formation is interpreted to represent continental deposition, with depositional characteristics comparable to those observed in the Eastern Desert; therefore, this sandstone is most likely associated with a fluvial-alluvial depositional model. The most significant oil–water contact (OWC) is identified at − 10,496 ft TVDss based on the GS173-2 well. However, indications from other wells within the study area suggest a potential upward revision of the OWC to − 10,318 ft TVDss. Oil gravities range from 14 to 39° API. Hydrocarbon generation is primarily attributed to the Campanian Brown Limestone and the Eocene Thebes (Radwany) formations, with additional geochemical indications supporting a Lower Rudeis source-rock contribution to the October D area^26^. The structure-contour map shown in **(**Fig. 3**)** delineates the wells incorporated into the static-model update. Cumulative production from the Nukhul sandstone reservoir in the October D area is approximately 18.5 MMBO^26^. The Nukhul Formation is of Lower Miocene age and corresponds to the T10 unconformity surface. It unconformably overlies the pre-Miocene section, which begins with the Thebes Formation at the T00 unconformity and continues downward to the granitic basement, and is overlain by the Miocene succession ranging upward from the Rudeis Formation to the Zeit Formation **(**Fig. 2**)**. Typical reservoir parameters derived from cored wells include porosity values of 13–19%, permeability of 340–640 md, and net-pay thicknesses ranging from 70 to 180 ft. The structural-contour map reflects the most recent structural update and mapping refinements for the area.

## Methodology

### Materials

The dataset used in this study consists from 20 seismic lines provided by the Egyptian General Petroleum Corporation (EGPC) through Gulf of Suez Petroleum Company (GUPCO) covering the area of study within October oil Field, in addition to five stratigraphic and structural control wells GS172-3ST1, OCT-D4, OCT-D1, GS173-2 and OCT-D5A **(**Fig. 3**)**. The materials contain the selected seismic lines along the area of study and a set of E-Logs that includes Caliper logs (CALI), Gamma ray logs (GR), Resistivity logs (RD), Density logs (RHOB), Neutron logs (NPHI), Sonic logs (DT), Dip-meter data, Core data from one well (GS173-2), Paleontological data, petrophysical evaluation for net gross and porosity calculations, Well test and production data. This study relies on both manual calculations and software tools, including Microsoft Excel, LAS editing tools, Canvas X 8.0, Techlog Software, Petrel, and the Schlumberger Chart Book (2005–2006).

### Workflow

**Fig. 4:**
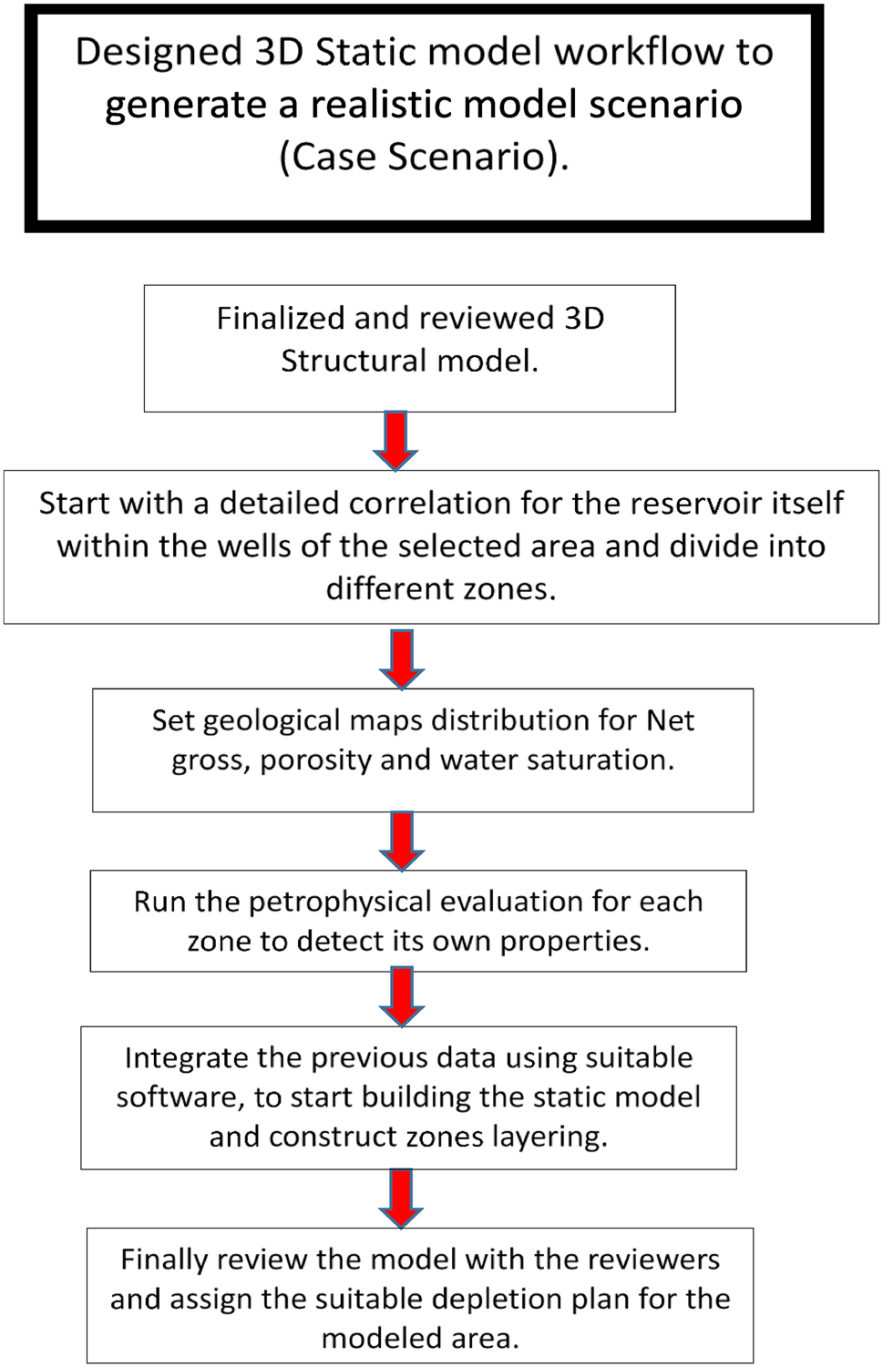
Flow chart of the integrated modelling work used in this study^5^.

The workflow employed in this study addresses the main structural and stratigraphic challenges of the area and outlines the procedures used to overcome these obstacles through an integrated approach using all available data **(**Fig. 4**)**. The datasets include detailed well-correlation data (ditch-cuttings descriptions, E-logs, dynamic data such as production and pressure), analogue outcrop interpretations, seismic sections, net-to-gross maps, porosity maps, saturation maps, and results from previous modelling efforts.

A key challenge in modifying the static model is reconciling and updating all available datasets—both historical and newly acquired—to produce a coherent and geologically realistic static model that supports opportunity identification and improves understanding of the reservoir system. Multi-source datasets were integrated into the 3D static modelling workflow^27^ through the following steps:

1. Collecting the previous work data: The first step is reviewing all earlier studies related to the area to establish a solid knowledge base and avoid starting from scratch.
2. Use the updated structural model: This step considers the foundation for the study, where adopt the most recent structural framework.
3. Apply geological knowledge: The next step is to use geological background, surface data such as out crops, and fundamentals to construct an initial play-scale interpretation that guides the modelling process.
4. Perform detailed well correlation: Detailed correlation for the selected wells especially on the target reservoir level to detect the reservoir geometry or facies change such as net-to-gross, porosity, shale content variation and water saturation (reservoir characteristics).
5. Define reservoir zones: Identify reservoir zones based on their reservoir characteristics, taking into consideration if the target reservoir already should be subdivided into more than multiple zones or treated as a single unit.
6. Integrate multidisciplinary data: The final step is to integrate the various disciplines and data in order to create an integrated product (Static model) and check the validity of new development opportunities, including the definition of a viable depletion plan when applicable.

### Methods

#### Data integration and reservoir modelling workflow

After finalizing and reviewing the structural model, petrophysical analyses, lithofacies maps, petrographic descriptions (from ditch cuttings and core data), and detailed reservoir-level correlations, multiple datasets were integrated to construct the final facies and static models^28^. The validity of dividing the reservoir into multiple zones was evaluated by examining sedimentological and diagenetic indicators derived from these datasets. The general description of the entire Nukhul Formation was based on ditch cuttings, thin-section samples, and core data analysis.

#### Stratigraphic correlation methodology

The stratigraphic correlation is based on five selected wells distributed across the area of interest to provide representative spatial coverage. Integration of regional geological understanding of the Gulf of Suez and the October Oil Field structural setting^17,19,22,29^, together with previous and current well correlation, structural modelling, and dynamic data, was used to interpret key stratigraphic markers within the Nukhul Formation. These markers form the basis for reservoir zonation from K1 to K4.

#### Petrographic analysis

Comprehensive reservoir petrographic analysis is essential for formation evaluation because it assists in identifying reservoir features and explaining differences in log response within the analyzed zones. The fundamental goal of the petrographic description of the Nukhul Formation is to characterize the basic lithology, matrix type, primary and accessory minerals, cement types, visual porosity, and hydrocarbon indications. Petrographic analysis was conducted to characterize lithology, matrix type, mineralogy, cement types, visual porosity, and hydrocarbon indications. Ditch cuttings and complete core from well GS173-2 samples were examined megascopically and microscopically using internal reports^30^ and reevaluated in this work. This analysis supports facies identification and interpretation of diagenetic controls on reservoir quality. The detailed description and variability within the Nukhul Formation, particularly the K4 zone is discussed using thin sections and core descriptions.

#### Facies modelling methodology

Two main techniques are commonly applied in facies modelling: (A) Sequential Indicator simulation, which creates a stochastic distribution of the property using variograms and data analysis **(REF)** and (B) Object-based modelling, which populates a discrete facies with different bodies of different geometries. In this study, object-based modelling was selected to represent Nukhul facies distribution based on core description and petrographic interpretation^31–35^. The following parameters are used to represent Nukhul facies distribution in the study area based on the core and surface understanding as the following:

o The facies body is Ellipse.
o Radial profile is Rounded.
o The orientation is Triangular.
o Minor width is Min is 150 m, the median/mean is 350 m and max is 550 m.
o Thickness is Min is 5 m, the median/mean is 20 m and max is 40 m.

Facies body geometry, orientation, width, and thickness parameters were defined from subsurface and surface analogs. Five major lithotypes—sandstone, conglomerate, shale, limestone, and basalt—were incorporated into the static model across zones K1–K4.

#### Static modelling parameters

The modified structural model and a zonation framework defined by stratigraphic correlation (K1–K4) were used to build the static reservoir model. Vertical layering was modified inside the K4 zone to capture high-frequency facies variability, and grid resolution was chosen to strike a balance between computational efficiency and the portrayal of geological heterogeneity. Petrophysical characteristics (porosity, net-to-gross, and shale volume) were conditioned to facies distributions and populated using well-log-derived data. Variogram ranges and item dimensions obtained from core observations, well correlations, and regional analogs were used to manage facies proportions and spatial continuity. Consistent depositional patterns at the reservoir size, facies stationarity within individual zones, and fault transmissibility controlled by qualitative fault seal analysis are among the model’s underlying presumptions.

## Results

### Stratigraphic correlation and reservoir zonation

#### Stratigraphic correlation

**Fig. 5:**
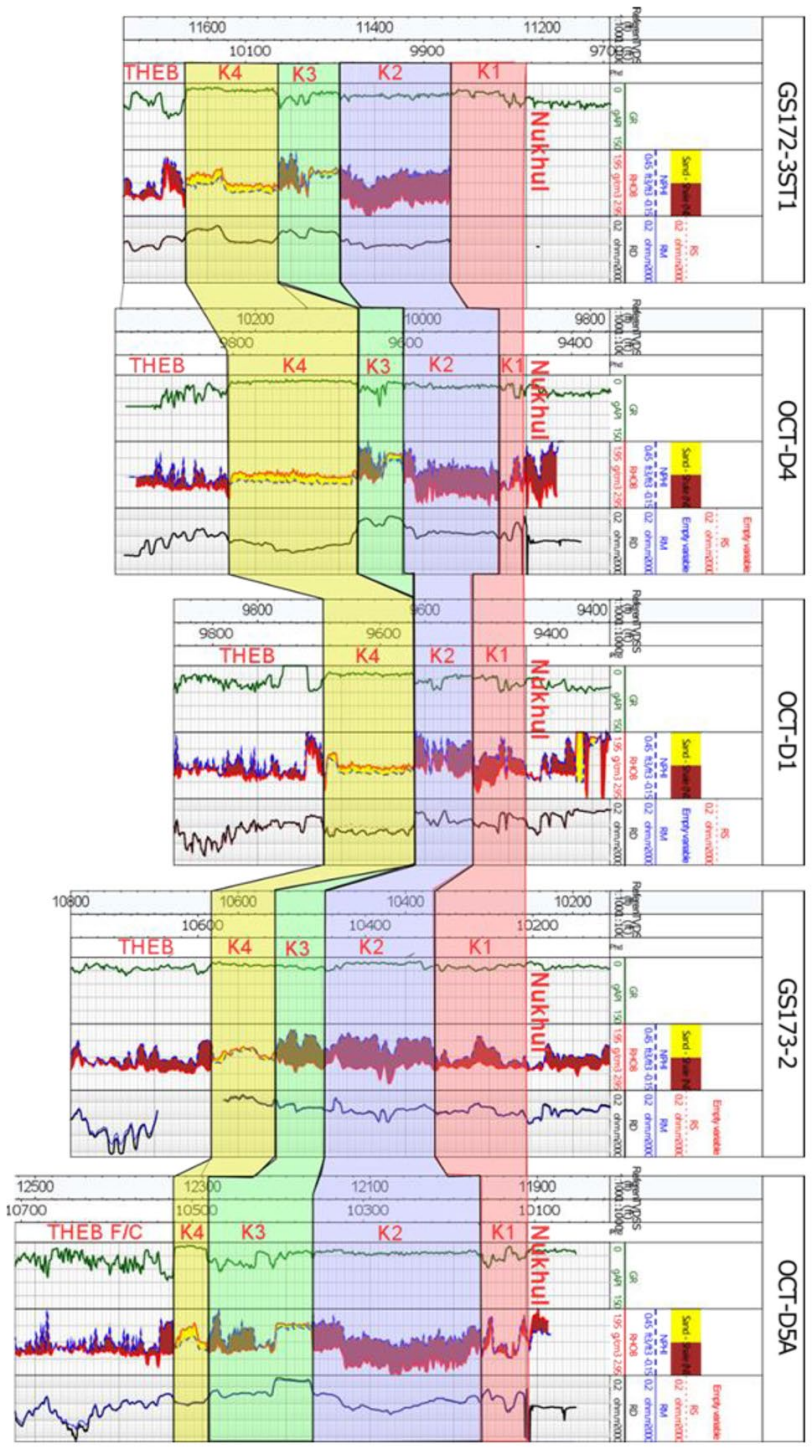
Subzonations of the Nukhul Formation into K1, K2, K3, and K4, based on the integration of core, log, and dynamic data.

The correlation is based on five selected wells. The wells are distributed across the area of interest to cover as wide an area as possible, providing more accurate and realistic results. Based on integration of the regional understanding of the Gulf of Suez and October Oil Field structural setting e.g.,^17, 19, 22, 29 and 36^, in addition to previous and current work including well correlation, structural modelling, and dynamic data, several markers were interpreted among the five wells along the Nukhul Formation. These markers define zonation from the top Nukhul (K1) to K4, with K4 being the main target reservoir due to its high-quality sandstone, wide lateral extent, and high productivity **(**Fig. 5**)**. This detailed correlation step is crucial for determining whether different characteristics within the formation justify defining additional reservoir zones.

The selected wells are sufficiently representative to support subdivision of the formation into K1, K2, K3, and K4 zones based on their characteristics. The wells contain a complete set of E-logs and ditch-cutting samples, and one well includes core material. These datasets are essential for updating and refining the static model.

#### Reservoir zonation and petrophysical characteristics

The reservoir characteristics and petrophysical data of the subzonations K1, K2, K3, and K4. The detailed characterization is as follows:

o The first well in this correlation is GS172-3ST1, which hit the complete section of Nukhul Formation as K1, K2, K3 & K4. Petrophysical properties for the whole formation are: net-to-gross 0.31, porosity 0.16, water saturation 0.45, and volume of shale 0.04. For K4: net-to-gross 0.98, porosity 0.14, water saturation 0.50, and volume of shale 0.04.
o The second well is OCT-D4, which has a missed section within K2 & K3 zones but away than the target reservoir K4. Whole-formation properties: net-to-gross 0.39, porosity 0.13, water saturation 0.20, and volume of shale 0.06. K4 properties: net-to-gross 0.97, porosity 0.11, water saturation 0.24, and volume of shale 0.05.
o The third well is OCT-D1, which has a missed section within K2 & K3 zones but away than the target reservoir K4. Whole-formation properties: net-to-gross 0.37, porosity 0.12, water saturation 0.24, and volume of shale 0.07. K4 properties: net-to-gross 0.94, porosity 0.12, water saturation 0.26, and volume of shale 0.07.
o The fourth well is GS173-2, which hit the complete section of Nukhul Formation as K1, K2, K3 & K4. Whole-formation properties: net-to-gross 0.25, porosity 0.15, water saturation 0.90, and volume of shale 0.19. The K4 zone lies down-dip and near the oil–water contact, leading to elevated water saturation: net-to-gross 0.97, porosity 0.16, water saturation 0.96, and volume of shale 0.11.
o The fifth well is OCT-D5A, which hit the complete section of Nukhul Formation as K1, K2, K3 & K4, while the target reservoir is very thin which will be explained later based on the net gross maps and facies model. Whole-formation properties: net-to-gross 0.16, porosity 0.19, water saturation 0.26, and volume of shale 0.11. K4 properties: net-to-gross 0.94, porosity 0.18, water saturation 0.26, and volume of shale 0.03.

### Petrographic description

**Fig. 6:**
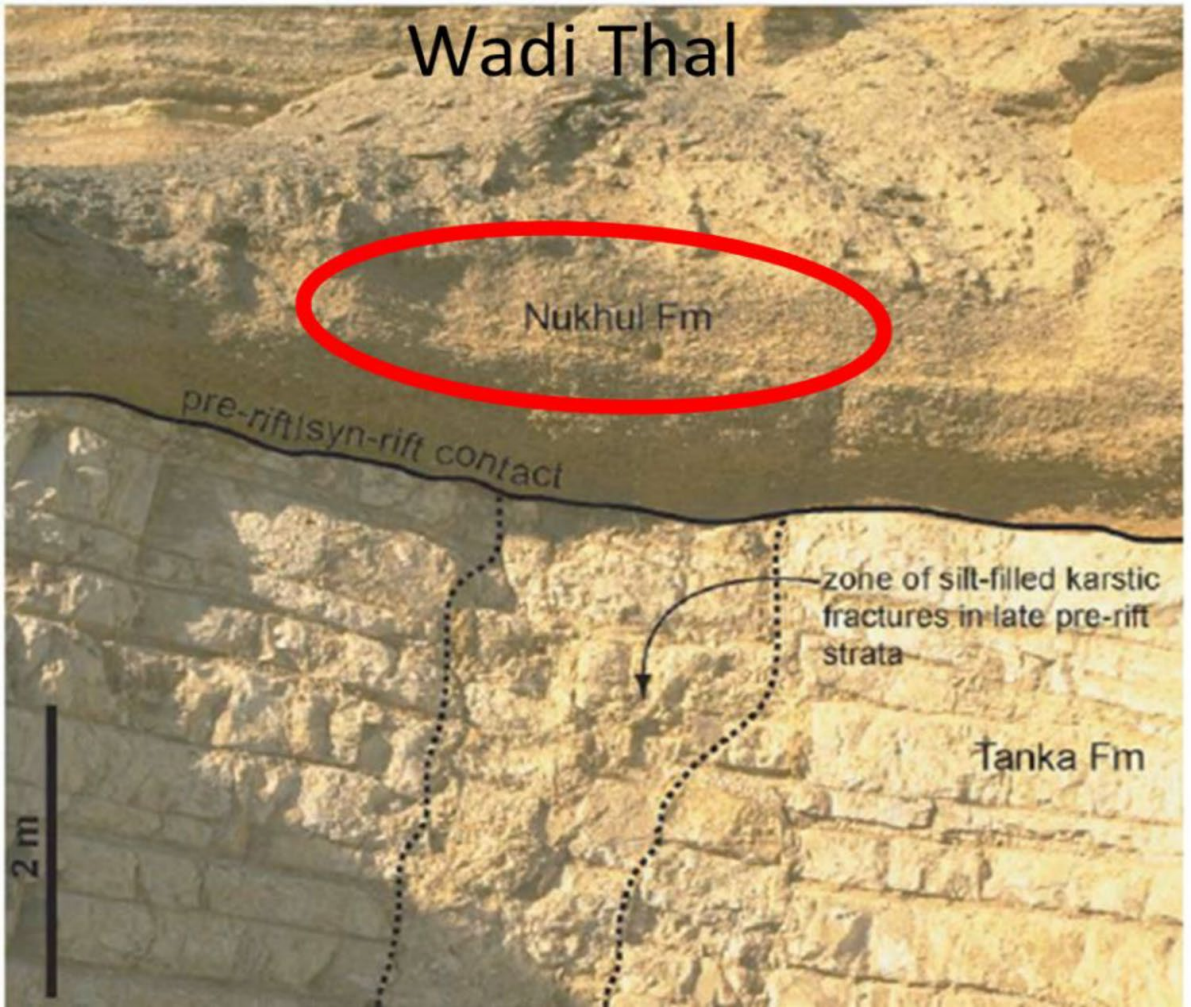
Surface geology of the Nukhul Formation (Wadi Thal), comparable to the subsurface Nukhul Formation within the study area^37^.

The Nukhul Formation, as outlined in the detailed correlation **(**Fig. 5**)**, and its subdivisions within the stratigraphic succession of the October Oil Field, are comparable to the surface geology of Wadi Nukhul, Wadi Thal, and Wadi Tayiba **(**Fig. 6**)**^37^. The main factors influencing the Nukhul Formation and resulting in its subdivision include the presence of different lithologies (clay minerals, limestone bodies, sandstone, and conglomerate), variations in cement types along the section, changes in log response, silica overgrowths within pore spaces, and the effects of compaction. The Nukhul petrographic description includes ditch cutting and core sample observations as the following:

#### Ditch cutting

Based on the ditch cuttings from the selected wells and visual estimates:

- Sandstone: colorless, loose, fine grained, occasionally medium grained, sometimes being conglomerate, rounded to subrounded, poorly sorted and in some parts is moderate sorted, porosity range 11–18%, the sandstone is mature, consists primarily of quartz grains.
- Shale: tannish grey, light grey, grayish white, blocky to subflaky, soft to moderate soft, occasionally high calcareous.
- Limestone: tan, brown, dark tan, occasionally light brown, buff, moderate hard to hard, argillaceous, rarely glauconitic and pyritic, with no visible porosity.

#### Core description

**Fig. 7:**
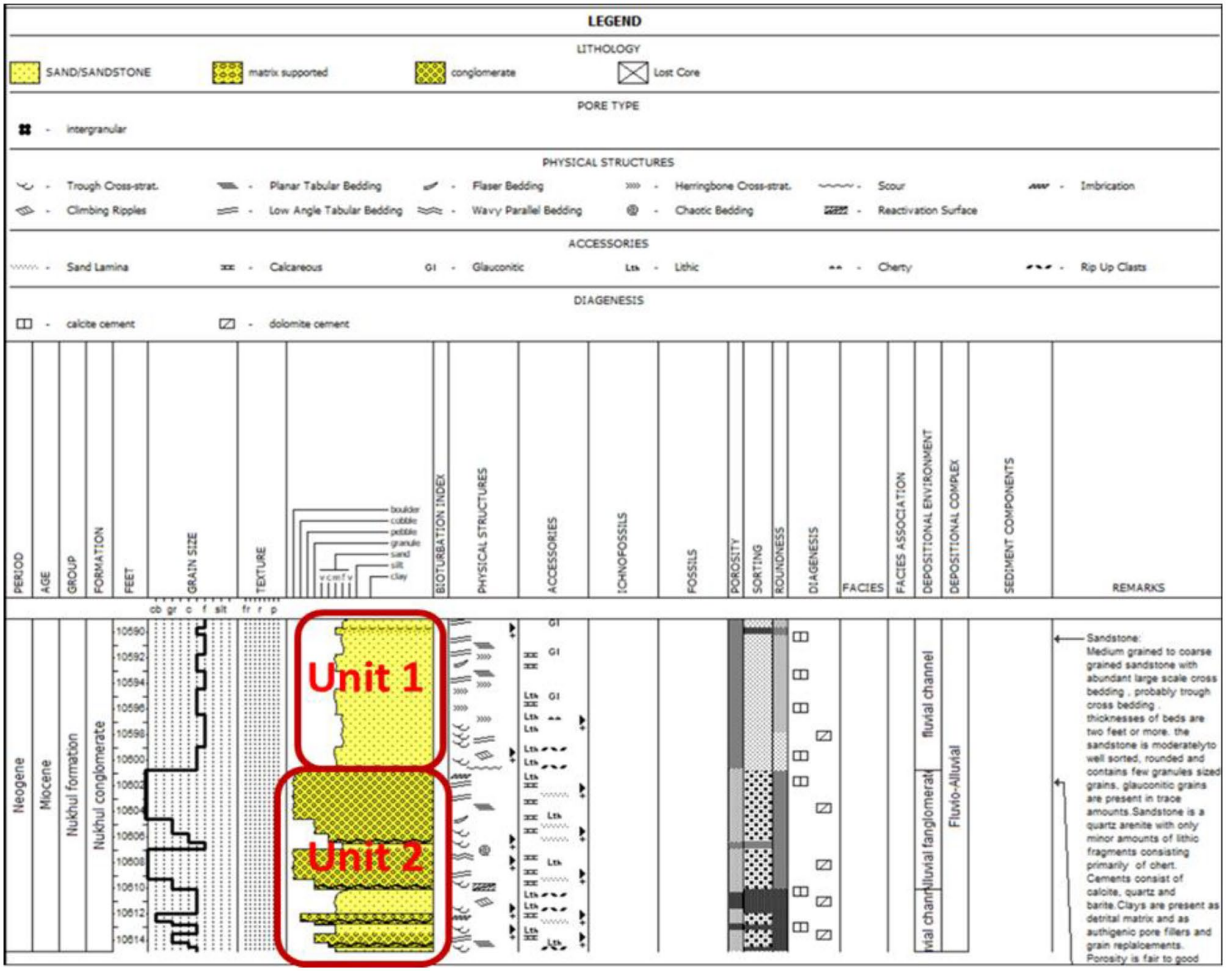
Nukhul reservoir core description as two different units (Unit 1 mainly sandstone & Unit 2 mainly conglomerate), depth interval 10,590 – 10,611 ft md.

**Fig. 8:**
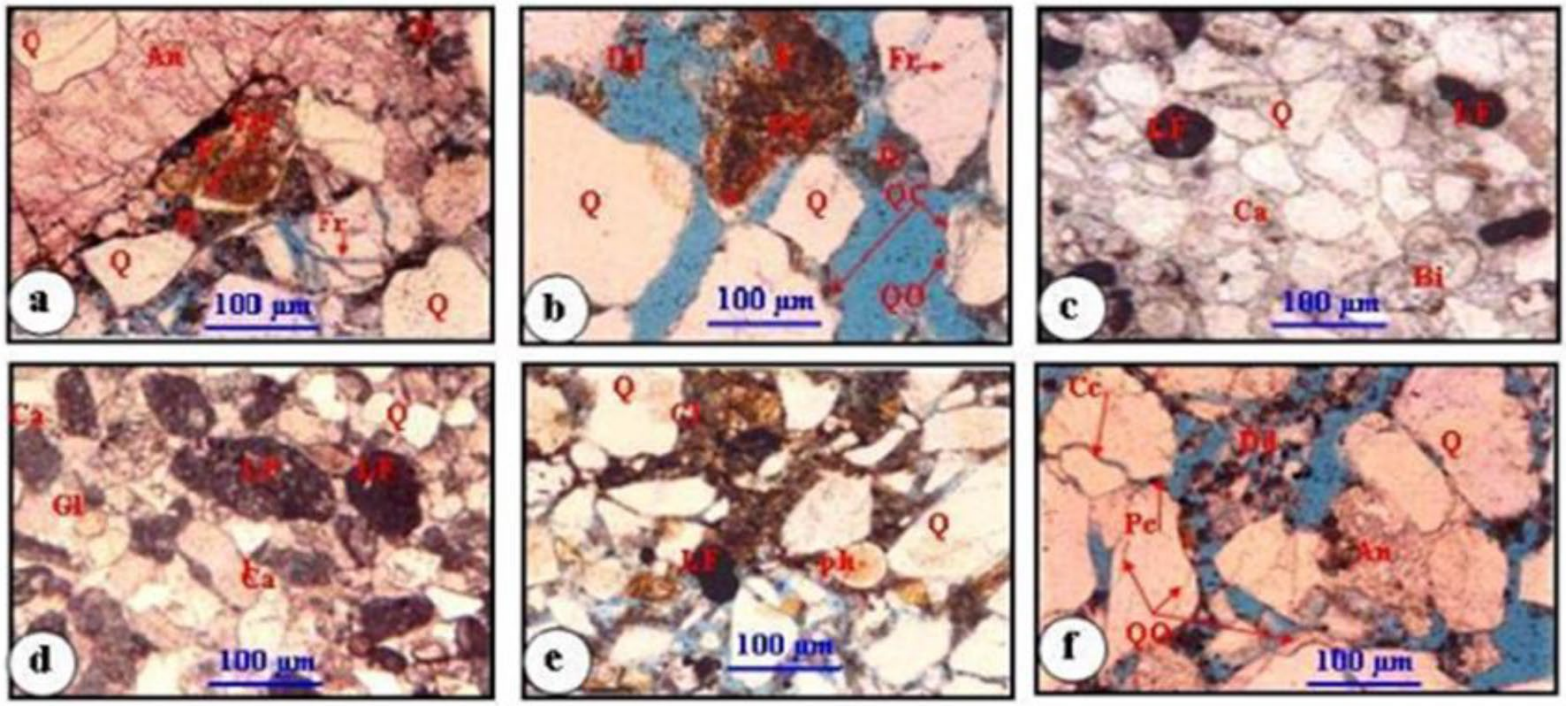
Photomicrographs of Nukhul sandstones showing: (a) Quartz arenite, detrital quartz and feldspar grains cemented by very coarsely crystalline anhydrite and micro to very finely crystalline dolomite (Note, dolomite cement engulfs feldspar overgrowth and engulfed by anhydrite cement, thus postdate feldspar overgrowth and predate anhydrite cement). (b) Quartz arenite, some of quartz grains show multiple deformation fractures, and quartz overgrowths engulfed by dolomite cement (Note, leached dolomite cement, and k-feldspar overgrowths). (c) Sublithic arenite, quartz grains are fine to medium -grained, sub-rounded to subangular and cemented with sparry calcite cement. (d) Lithic arenite, medium to coarse lithic fragments and glauconite are scattered in calcite cement (Note, detrital quartz coated with iron oxide cement that engulfed by calcite cement, thus predate calcite cement). (e) Sublithic arenite, very poorly sorted sandstone with a patchy detrital clay matrix replaced by dolomicrite cement, (Note, the well-rounded fragment of bone). (f) Quartz arenite, dissolution of microcrystalline dolomite (Note, point- long and concave-convex contacts, anhydrite cement and quartz overgrowth).

General lithological units, rather than depositional units, were identified and described individually on graphic core logs and correlated with well logs **(**Fig. 7**)**. Raw data were derived from^30^. Diagenesis includes the physical, biological, and chemical changes affecting sediments after deposition and prior to metamorphism^38^. Lithologies with excellent primary intergranular porosity **(**Figs. 8b and 8f**)** and permeability may be altered by cementation and compaction during diagenesis **(**Figs. 8a, 8c, 8d, and 8e**)**. The cored Nukhul reservoir consists of medium-grained to pebbly sandstone with fair to good porosity. The reservoir exhibits probable lateral continuity. Compositionally, the sandstones are quartz arenites with minor lithic fragments. Cementation is dominated by quartz overgrowths with patchy calcite spar and barite cements. The cored samples contain no major permeability barriers within the same zone. One of the main objectives of coring was to delineate reservoir zonation within the Nukhul Formation and to identify major textures and the relationship between diagenesis and reservoir properties.

#### K4 zone Unit1 (10,590’ – 10,600‘)

Consists of four cross-bedded, medium-grained sandstone beds **(**Fig. 7**)**. The only mudstone present within the cored interval consists of mud clasts within the pebbly sandstone beds.

#### K4 zone Unit2 (10,600.5’—10,614’)

Sandstone is medium to coarse-grained with abundant large-scale cross-bedding, probably trough cross-bedding **(**Fig. 7**)**. Individual beds are two feet thick or more. The sandstone is moderately to well sorted and rounded, containing minor glauconite. A clay-rich sandstone layer is present, but its lateral extent is likely limited. The sandstone is a quartz arenite with minor lithic fragments composed primarily of chert. Cements include calcite, quartz, and barite. Clays occur as detrital matrix and as authigenic pore-fill and grain-replacement phases. Porosity is fair to good (11–18%) and includes secondary intragranular, primary intergranular, and microporosity.

The conglomerate within this interval consists of coarse-sized pebbles and boulders of chert and flint, moderately to poorly sorted, and shows slight imbrication at the tops of cycles. The cement is most likely calcareous. **(**Figs. 7 and 8**)** The conglomerate is typically polymictic and displays high hydrocarbon content, with heavy oil or asphaltene commonly observed at the basal parts of cycles. **(**Fig. 7**)** supports the interpretation that the reservoir should not be treated as a single bulk unit but instead subdivided into multiple units. On the other hand, **(**Fig. 8**)** is supporting that the reservoir should not be taken as a bulk and should be divided to different units, that will be discussed in detail through the detailed description for each unit.

#### Lithofacies maps and fault seal analysis

Based on the different characters within the whole Nukhul Formation such as sand and shale distribution, thickness variation, porosity differentiation, and volume of shale variation, there was a need to divide the Nukhul Formation into different zones as shown in (Fig. 5). This idea will be clarified by showing the net-to-gross, porosity, and shale volume maps that will be integrated to serve the facies model, which is essential for initiating the depletion plan to identify the best locations for adding appraisal, exploratory, and development opportunities. Any idea of new opportunities will start with structure or compartmentalization, attic locations, reservoir extension, and facies variation to detect reservoir enhancement locations. Based on previous and current work, the conclusion is to work on the K4 zone as the main target of interest due to because of its highest oil production, while other zones are less important because of their poor quality and non-contribution to oil production. To confirm that there are promising opportunities, the trap and seal should be present and effective to assure oil presence and prevent escape from the reservoir, consequently the fault seal analysis exercise is critical for clarifying the sealing effect and the juxtaposition between reservoir and non-reservoir units.

#### Sand lithofacies maps.

**Fig. 9:**
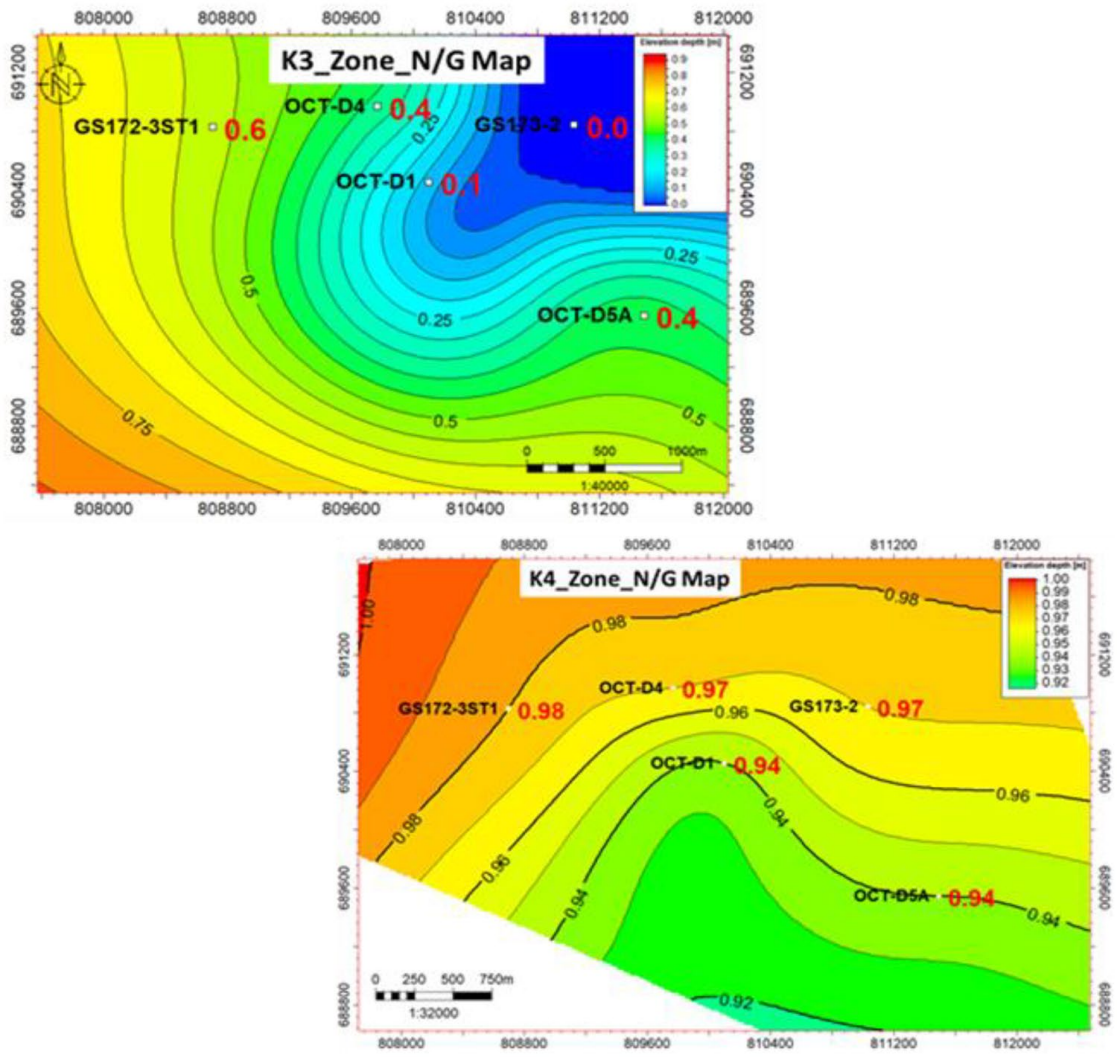
The sand net gross maps for K3 and K4 zones illustrate the changes in sand thickness and distribution direction, taking into considerations the offset wells in the background. The colors red, orange, and yellow indicate the direction of sand enhancement, while green, cyan, and blue indicate the direction of sand presence decrease, which primarily affects the selection of future opportunities.

As seen in the detailed correlation and production history data, the main target reservoirs for optimum productivity are the K3 and K4 zones in this study, which makes their precise detection critical for a better development plan. Constructing sand net-to-gross maps is beneficial for defining reservoir presence, distribution, and extension, as well as identifying the optimum target areas for development planning. Therefore, it was necessary to examine all related wells and others in the background to achieve the best findings with minimal uncertainty. The net-to-gross map in (Fig. 9) shows sandstone distribution and identifies sandstone enhancement locations, where the northwest direction is the area of sandstone increase as shown in the legend (dark colours such as red and brown indicate increasing sandstone thickness, while light colours such as yellow, green, and blue indicate decreasing sandstone thickness). Therefore, the best locations for sandstone enhancement are to the north of OCT-D4 and GS172-3ST1, as preliminarily concluded from the sandstone net-to-gross map.

#### Shale lithofacies maps.

**Fig. 10:**
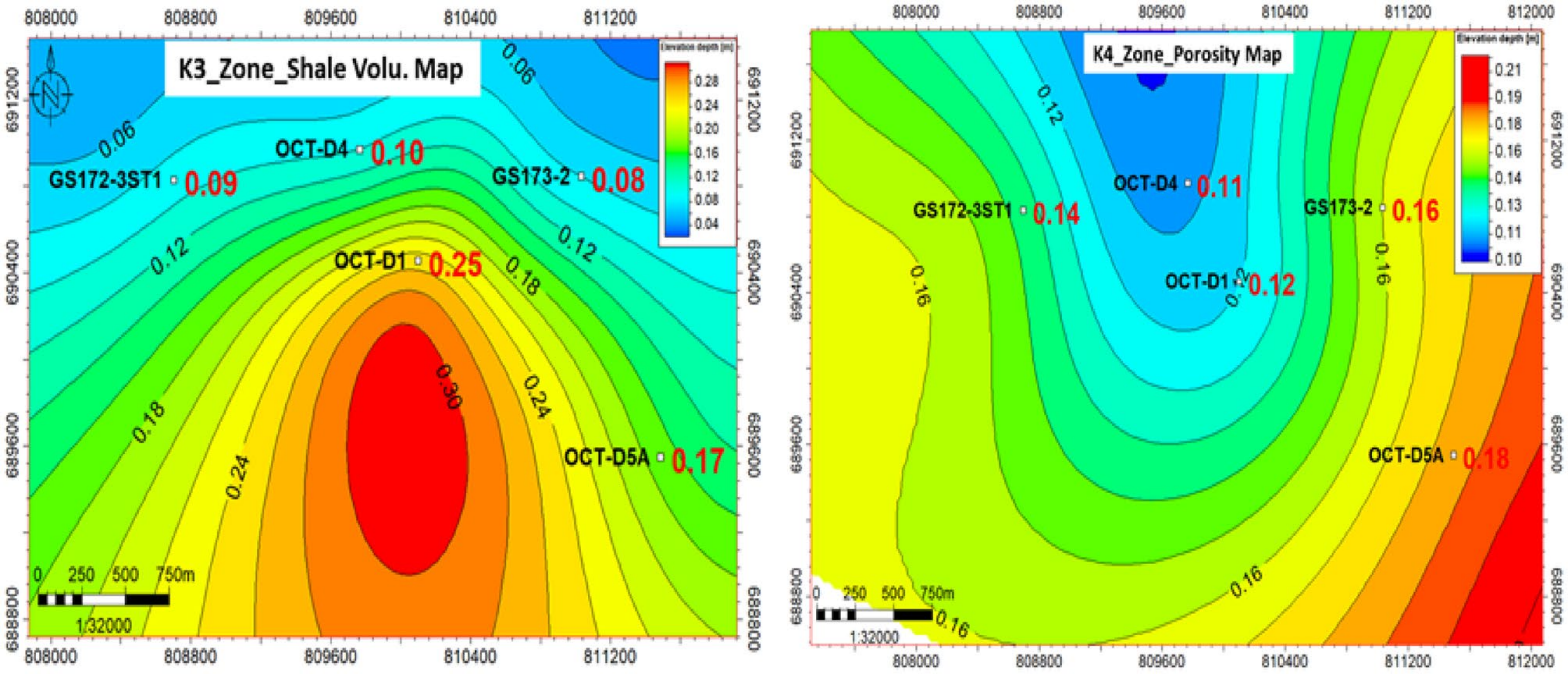
K3 and K4 zones shale volume distribution maps show variations in shale content while accounting for offset wells in the background. According to the interpretation of those maps, the dark colors red, orange, and yellow indicate the direction of shale growth, which negatively affects the improvement of the facies, while the green, cyan, and blue colors indicate the direction of shale amount decrease, which improves the location of future opportunity selection.

One of the main factors controlling the facies model is the shale volume distribution, as any target opportunity should be located away from the shale increase due to its negative effect on permeability, which reduces oil migration and productivity enhancement. As shown in (Fig. 10), shale volume variation is clear, where the northwest direction of OCT-D4 and GS172-3ST1 represents a zone of decreasing shale volume, in addition to the southern area near OCT-D5A well. The attached legend shows that dark colours represent increased shale volume, which decreases the chance of adding an opportunity in those locations, so it is preferred to move toward the light-colour regions.

#### Porosity maps

**Fig. 11:**
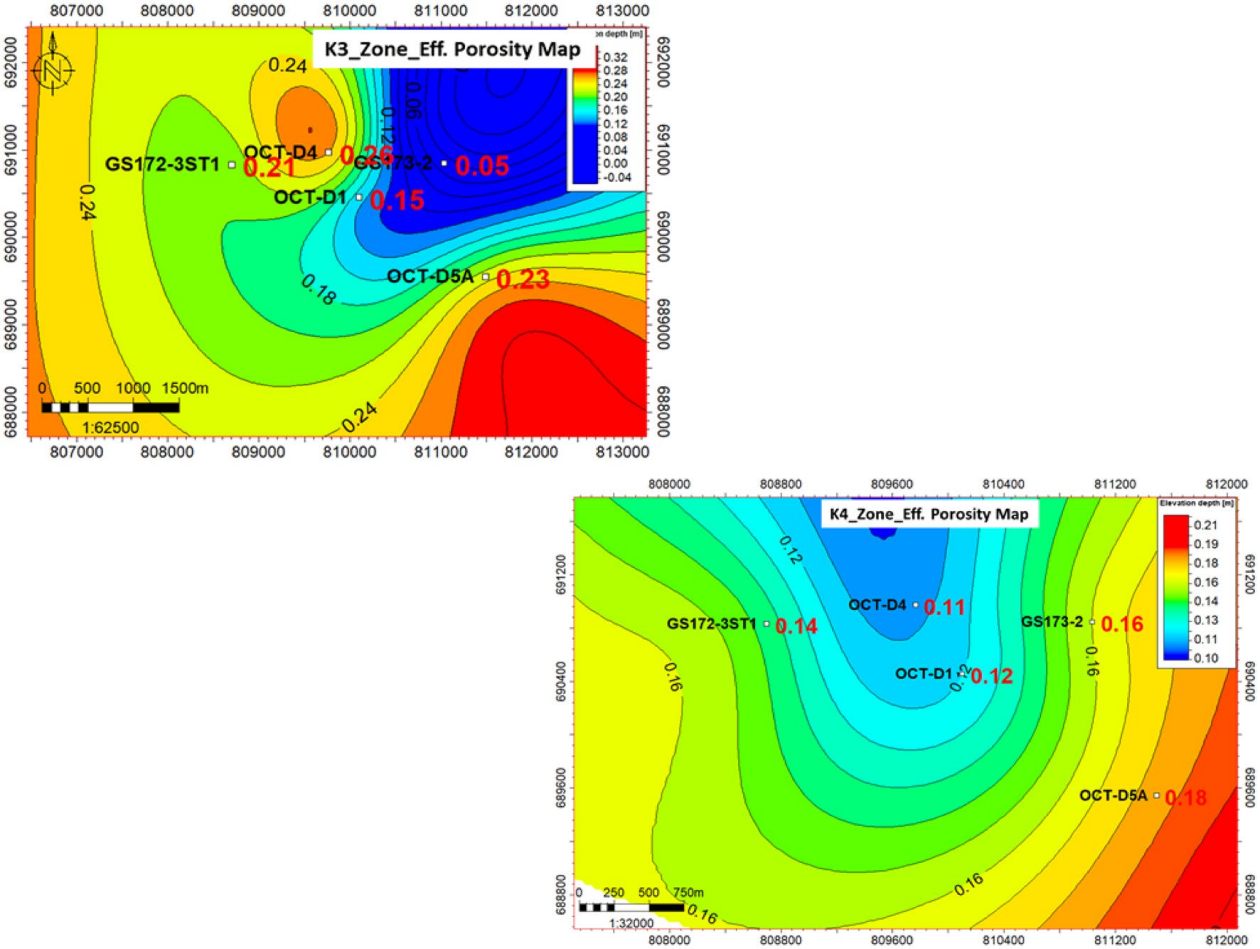
Effective porosity distribution map for the K3 & K4 zones, showing the porosity enhancement in the northwest and southeast directions toward GS172-3ST1 and OCT-D5A wells, rather than the east direction, which reflects the proposed location for the future opportunities if viable.

Porosity is an essential factor with a direct impact on opportunity value, so it is recommended to search for high-porosity locations. Porosity is one of the input parameters for the static model. As shown in (Fig. 11), porosity varies across the area of interest, with enhancement toward OCT-D5A well and south of GS173-2 and OCT-D1 wells. The legend shows that dark colours represent increased porosity, improving the chance of adding opportunities in these locations. By combining the different elements described earlier, the static model can be initiated to represent facies distribution across the area of interest.

#### Fault seal analysis

**Fig. 12:**
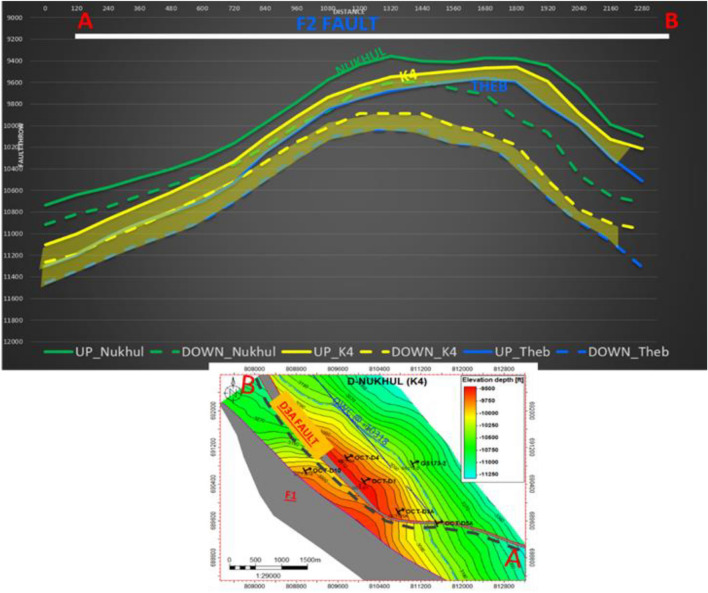
Fault seal analysis for F2 fault is qualitative to show the upthrown and downthrown for this fault that control the proposed locations for the next opportunities. The dashed lines are for the down thrown for the selected formations and zones (Nukhul Formation, K4 zone & Thebes Formation), while the solid lines are for the upthrown blocks. The fault seal analysis is very critical to clarify the sealing effect, and the juxtaposition between the sand reservoir and other lithologies such as shale and limestone.

The fault seal analysis for the F2 fault is qualitative—which controls the upthrown and downthrown blocks and has significant influence on proposed opportunities because of its critical location, as shown in **(**Fig. 12**)**—is essential for clarifying the sealing effect and the juxtaposition between reservoir and non-reservoir units. It is recommended to identify the optimal location for reservoir accumulation, trap integrity, and seal efficiency. This helps define the best oil production sites and supports constructing a better development strategy for the area.

To summarize, the extension and throw values of these faults play an essential role in oil reserve amounts, the entrapment system, and reservoir continuity with neighbouring reservoirs.

As a product of the F2 fault seal analysis:

K4 in Up-thrown Block is against the basal part of Mheiherrat (Lower Rudeis Member) & Nukhul LS in the Down-thrown.

K4 in the Down-Thrown is against Thebes carbonate in the Up-thrown.

The fault throw is around 250–300 ft in OCT-D3A well location.

### Rock facies & Facies model results

The reservoir characteristics of the sandstone and conglomerate interbeds are as follows:

1. Sandstone: the average permeability is 500–750 md, average porosity is 10–19%, grain-size sorting is good, grain size is medium, grain roundness is subrounded, the degree of consolidation is loosely consolidated, bedding is cross-bedded, and the type of cementation is carbonate.
2. Conglomerate: the average permeability is 100–500 md, average porosity is 10–19%, grain-size sorting is poor, grain size is very coarse, grain roundness is subangular, the degree of consolidation is well consolidated, bedding is massive, and the type of cementation is carbonate.

In general, the Nukhul reservoir (sandstone and conglomerate) is interpreted to represent continental deposition similar in style to that observed in the Eastern Desert; thus, this sandstone is most likely to correspond to a fluvial-alluvial fan model^17^.

The general description for the Nukhul Formation (K4 zone) from thin-section samples and core data analysis was described as shown in **(**Figs. 7 and 8**)**. Facies analysis or conceptual model is based on integrating all available data to produce a realistic scenario capable of justifying and clarifying all relevant issues in the area of interest that needs to be established. According to the interpretation of the facies analysis, five major lithotypes were identified within the Nukhul Formation: sandstone, limestone, shale, basalt, and conglomerate, where these facies lithologies are distributed along the model within the different zones from K1 to K4.

#### Facies model of K1 and K2 zones

**Fig. 13:**
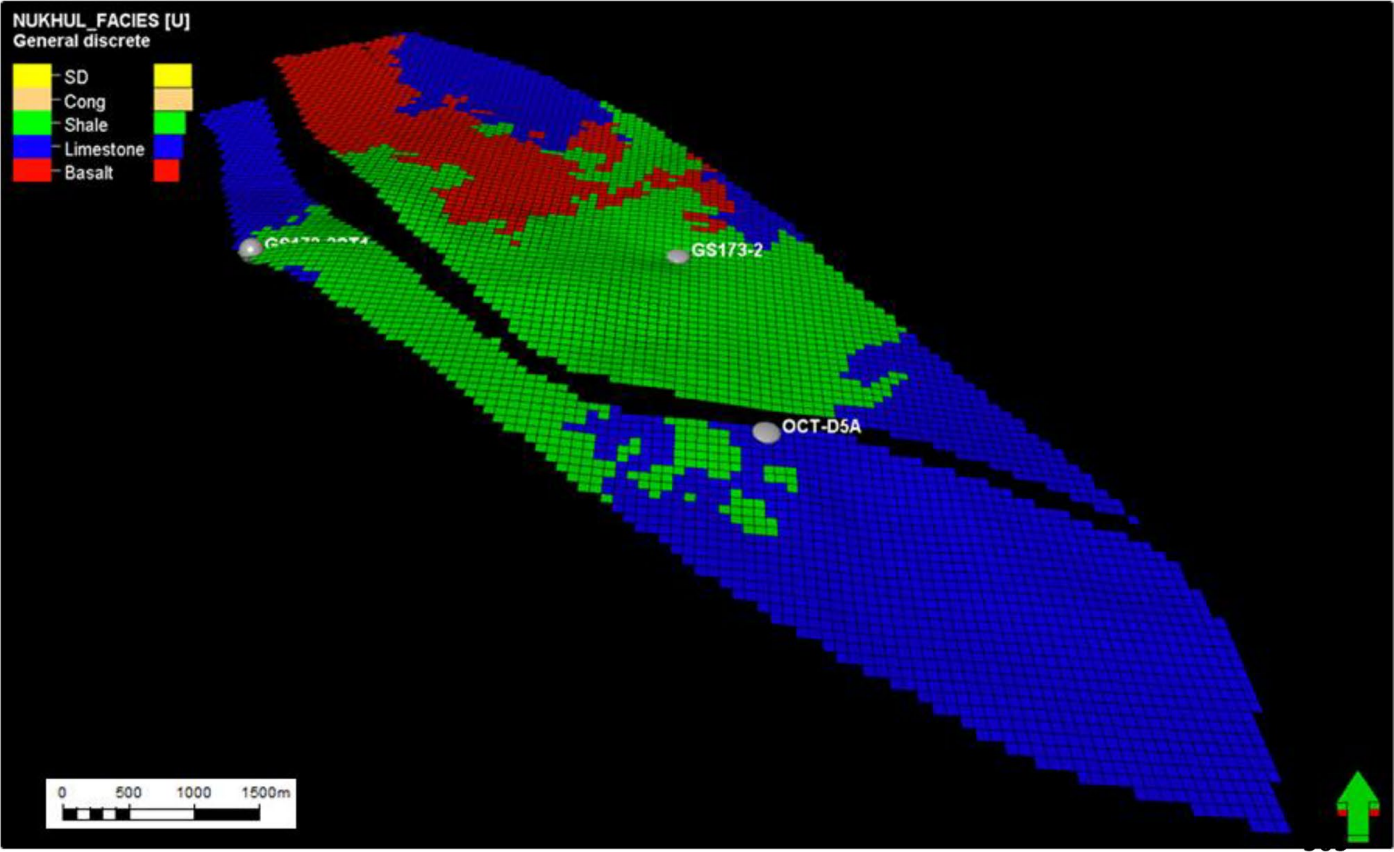
Nukhul layer 5 (K1 zone), indicating the general distribution for the different lithologies as shale, limestone, and basalt, which is proposed to act as lateral and vertical seal.

**Fig. 14:**
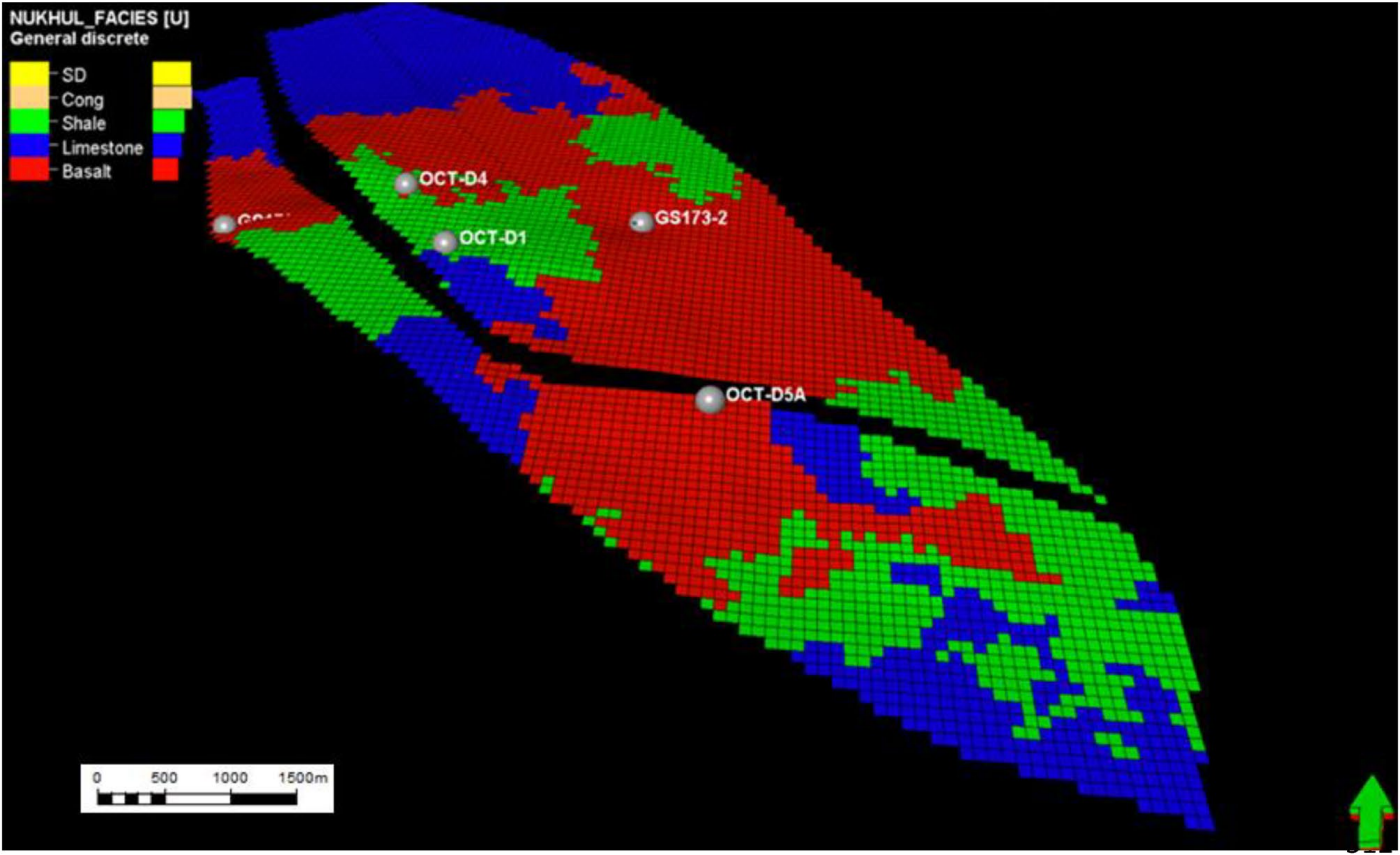
Nukhul layer 13 (K2 zone), indicating the general distribution for the different lithologies as shale, limestone, and basalt, with some indication of basalt percentage increase.

K1 & K2 zones are considered non-reservoirs, where their facies lithologies are mainly limestone, shale, and basalt in the absence of sandstone and conglomerate. These lithologies are identified through ditch-cutting thin sections, in addition to wireline logs. These two zones have a very important role in hydrocarbon accumulation, where they act as very good vertical and lateral seals because of their low porosity and permeability, which prevents hydrocarbon escape. This is clear in their distribution within the facies model across the area of interest shown in (Figs. 13 and 14).

#### Facies model of K3 zone

**Fig. 15:**
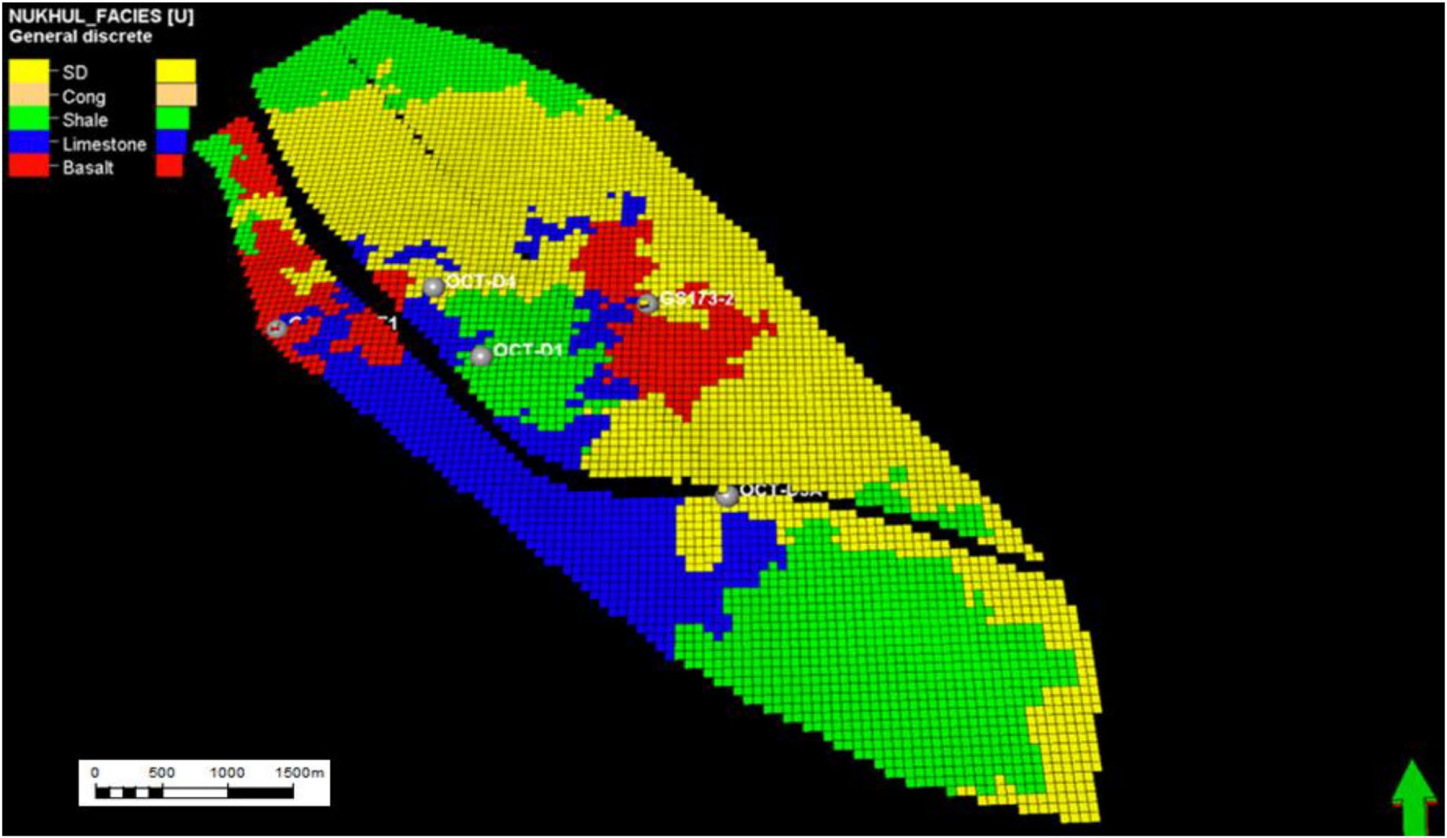
Nukhul layer 28 (K3 zone), indicating the general distribution for the different lithologies as sandstone, shale, limestone, and basalt.

**Fig. 16:**
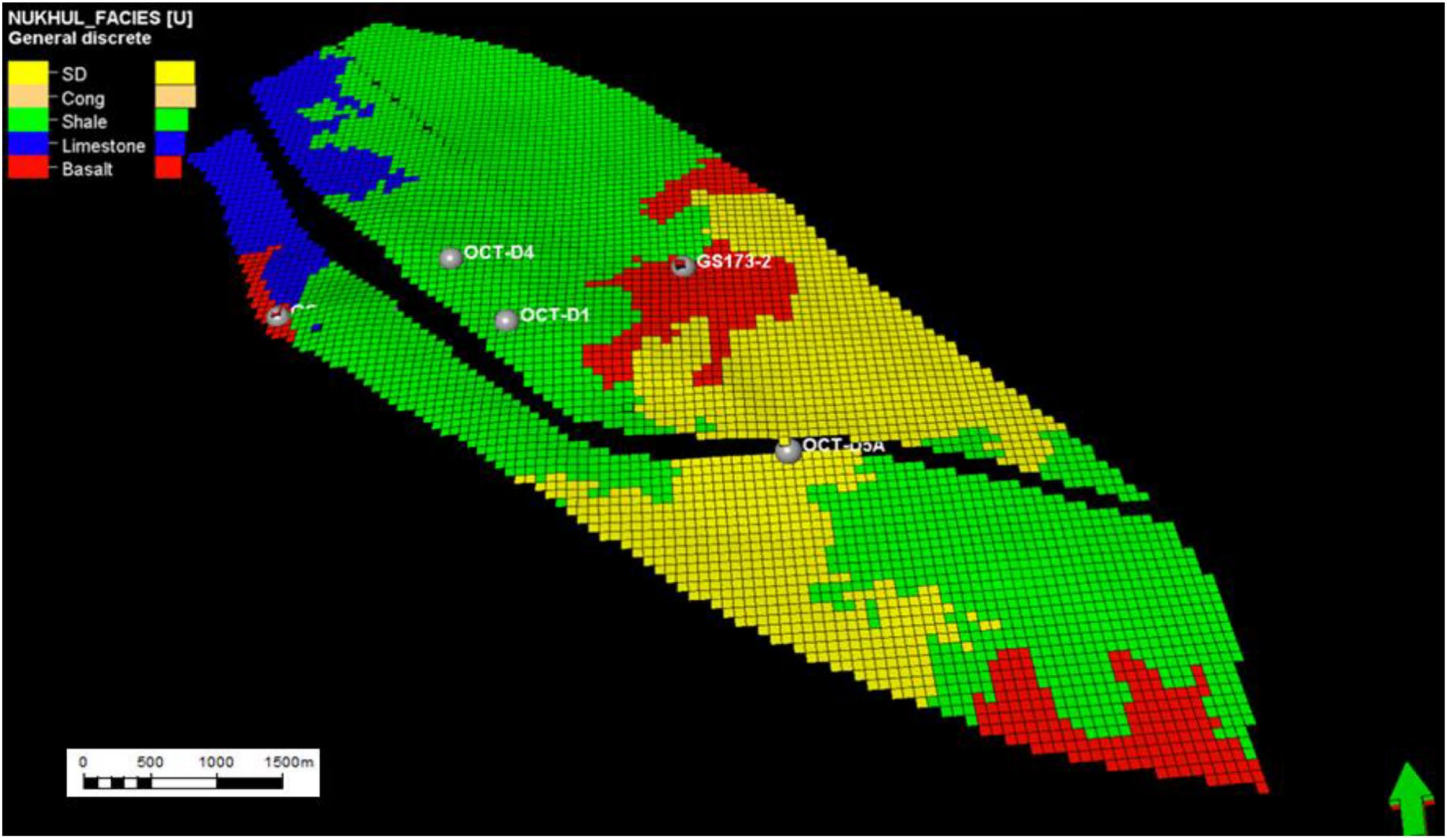
Nukhul layer 41 (K3 zone), indicating the general distribution for the different lithologies as sandstone, shale, limestone, and basalt without any indication for a specific trend for the sand distribution which reflects the difficulty of areal development through this zone.

As seen in the attached figures generated from the facies model, the K3 zone is regarded as a reservoir zone but differs from the K4 zone in being of lower quality, less extensive, and having thinner sandstone, as shown in (Figs. 15 and 16). Since the K3 zone is not regarded as one of the primary targets, one of the study objectives was to determine how to develop the region by adding the K3 zone as a target reservoir through determining the most effective method for sandstone distribution and extension detection. Four lithofacies were identified in the K3 zone from cored and thin-section samples, in addition to wireline logs: sandstone, shale, limestone, and basalt. The lack of a distinct distribution trend in the sandstone suggests that it is difficult to develop, which implies the importance of constructing the facies model, where its presence in this case is crucial.

#### Facies model of K4 zone

**Fig. 17:**
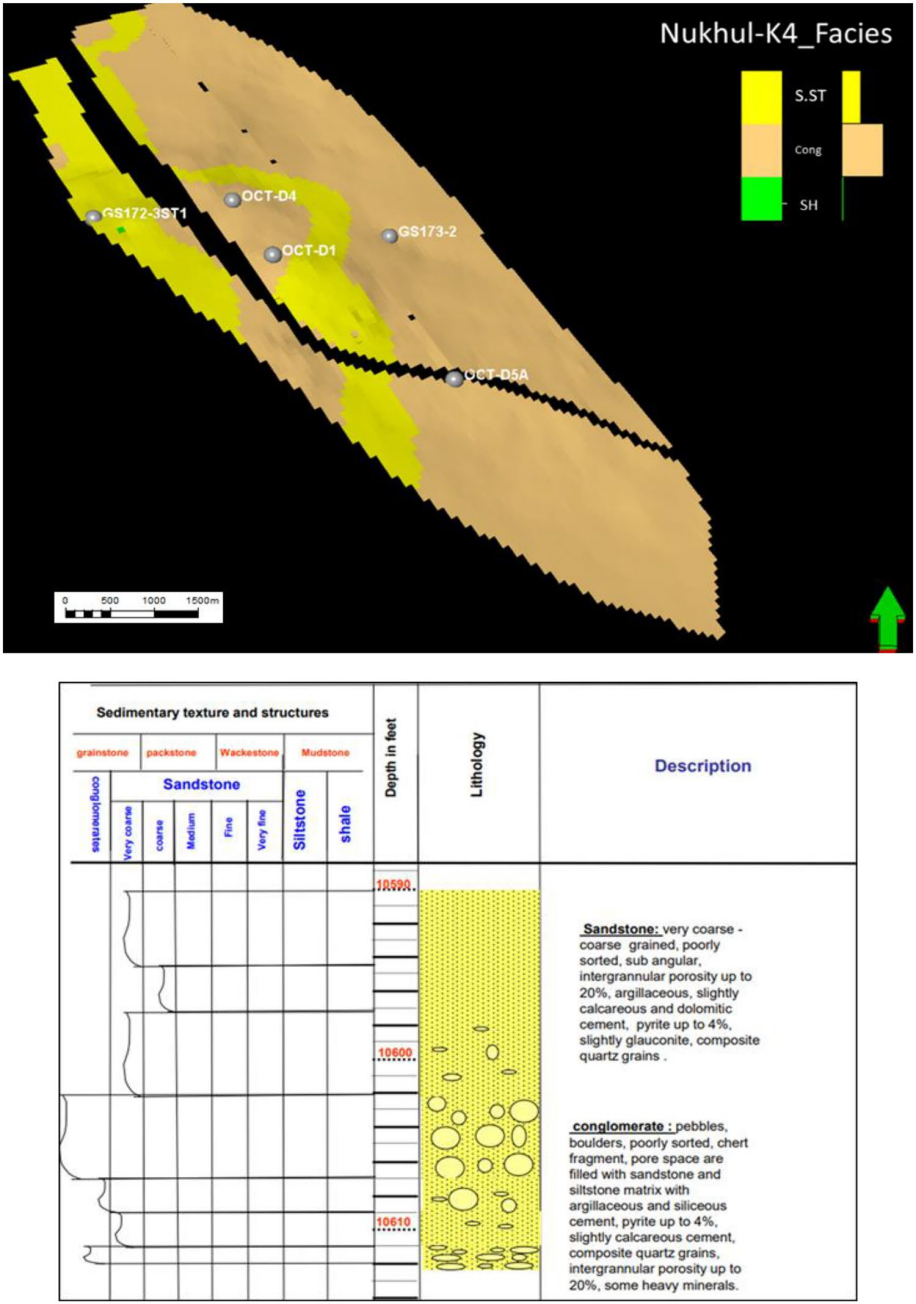
Nukhul sandstone layer 57, indicating the sandstone and conglomerate distribution and trend.

**Fig. 18:**
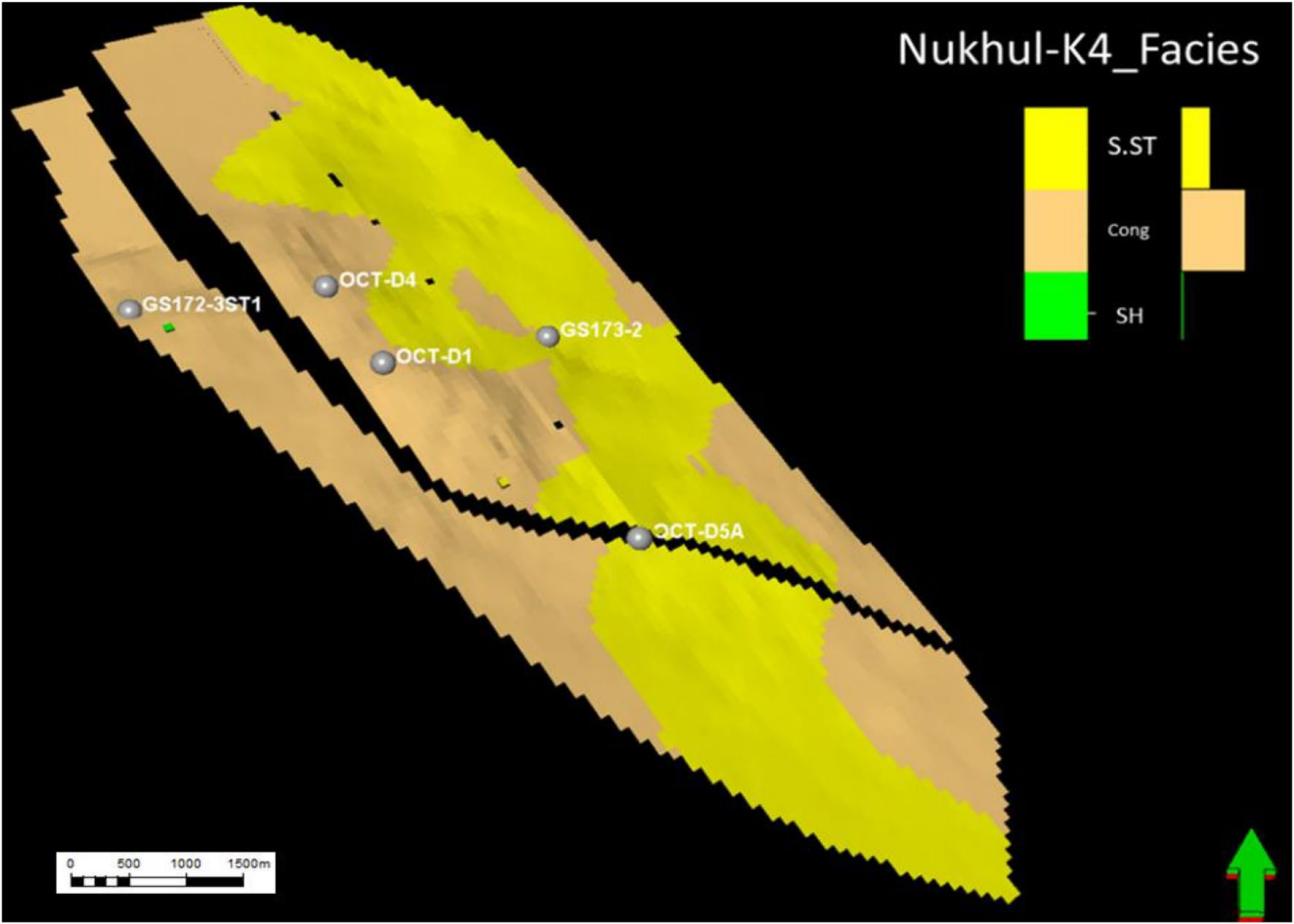
Nukhul sandstone layer 65, indicating the sandstone—conglomerate distribution variation and their trend.

**Fig. 19:**
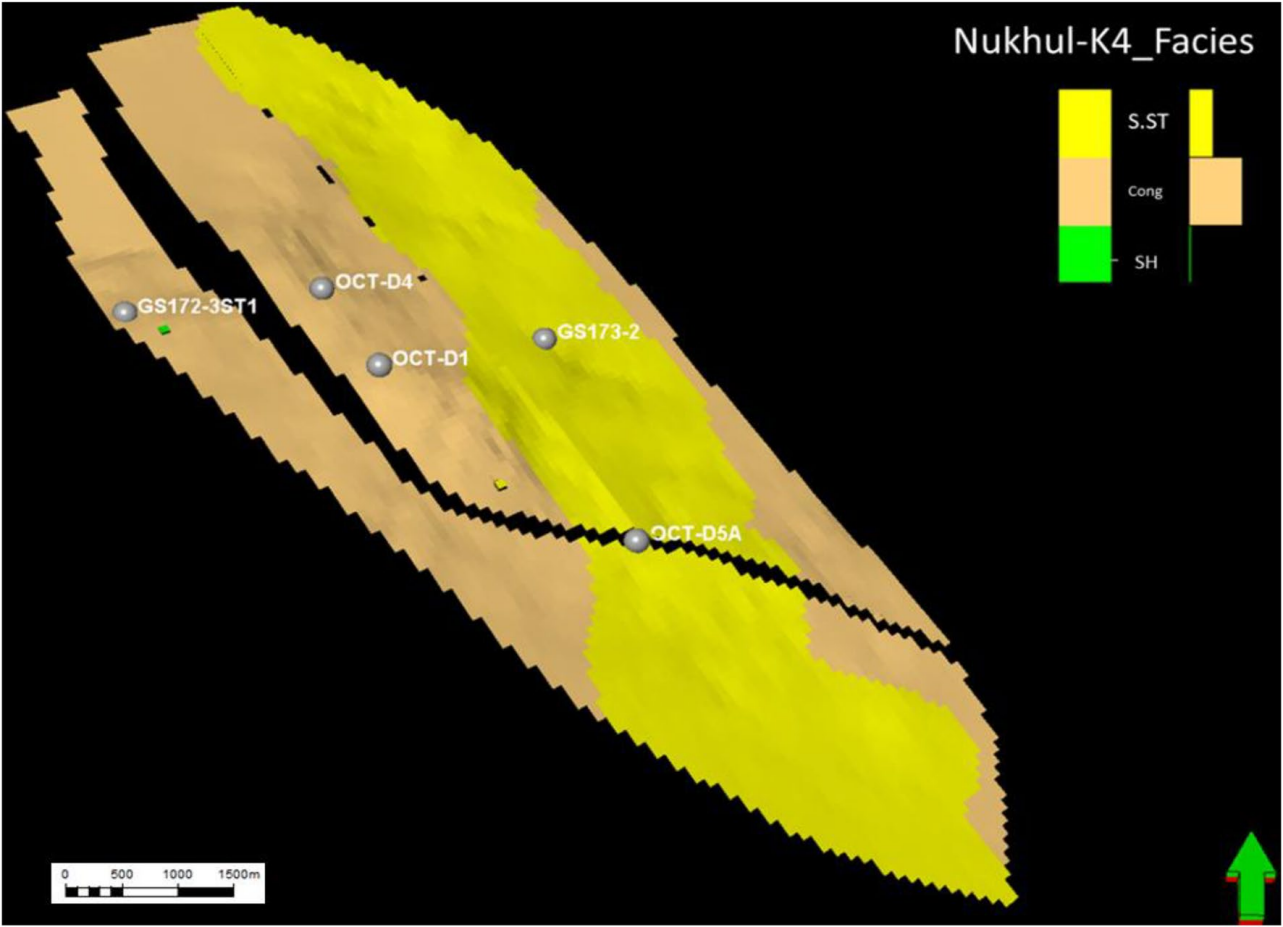
Nukhul sandstone layer 72, indicating the sandstone channel trend with large width and extension in the north – south direction.

**Fig. 20:**
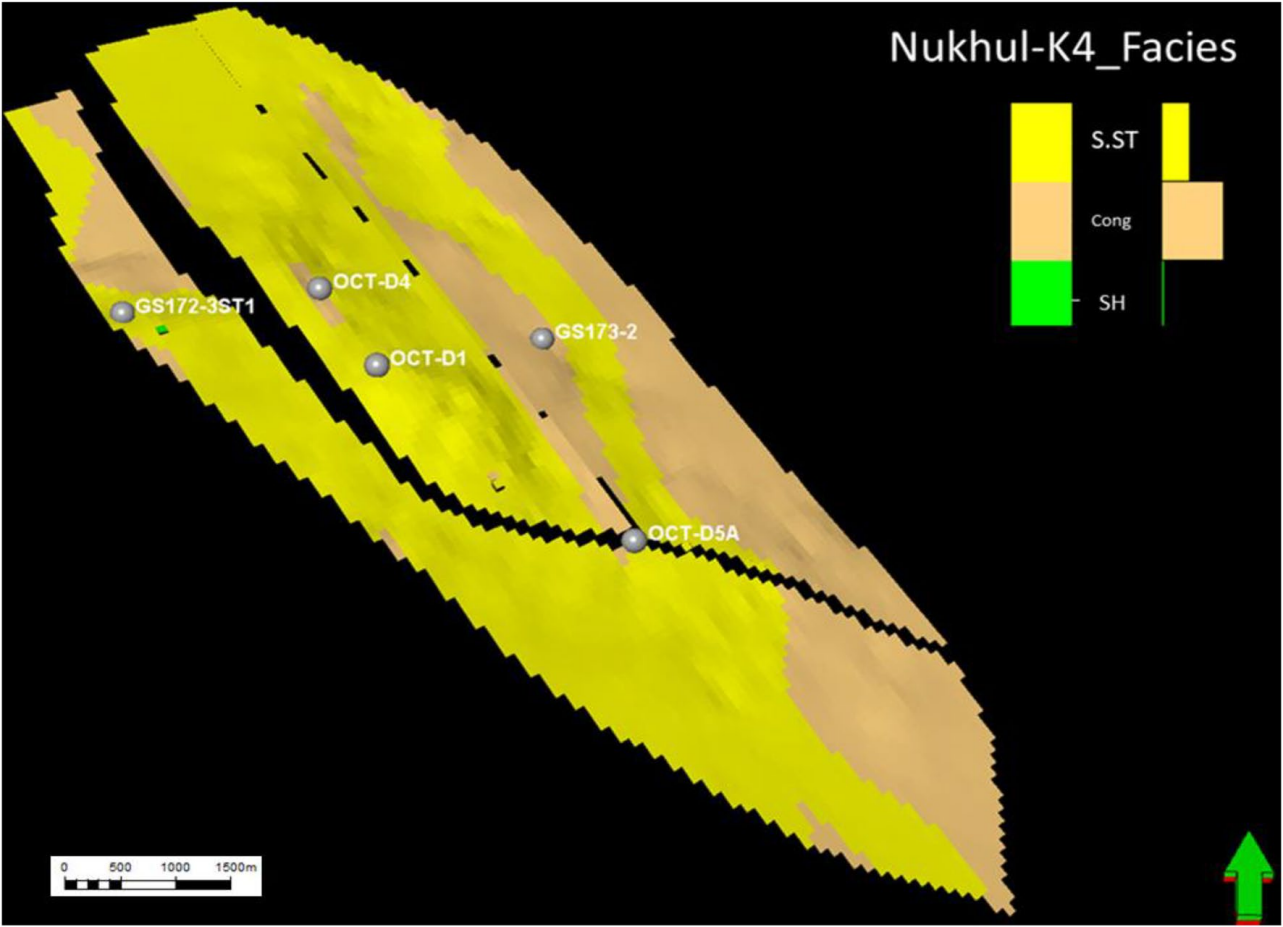
Nukhul sandstone layer 75, indicating the sandstone channels trend with large extension in the north – south direction, in addition to the well’s location within the sandstone channels.

As mentioned earlier, the K4 zone is the most important zone, having the greatest economic value in the oil production history of this study area. Three lithofacies were identified in the K4 zone from cored and thin-section samples: sandstone, conglomerate, and shale. Sandstone has the highest productivity rate compared with conglomerate, making it the most important unit. The message conveyed from (Figs. 17, 18, 19, and 20) is that the K4 zone shows high variation in sandstone, conglomerate, and shale trends, where across the layers of the static model, sandstone enhancement occurs in certain directions and locations. The static model for the K4 zone was divided into 30 layers, from layer 51 to 80, to define the intricacies of facies changes within this reservoir interval. Figure 17** (**Fig. 17**)** along layer 57 demonstrates that the predominant component in this area is conglomerate, which is considered the background, while the sandstone channel (target reservoir) trend is north–south with some sinuosity. Based on the attached log in **(**Fig. 17**)**, the conglomerate is the dominant lithotype, making up more than 70%.

Figure 18 along layer 65 shows good development of sandstone percentage, width, and direction. Based on thin-section description, sandstone percentage reaches nearly 50%. The sandstone channel trend remains north–south with increased sinuosity.

Figure 19 along layer 72 shows further development in sandstone percentage, width, and direction. In addition, there is a decrease in channel sinuosity, forming a wide and extended sandstone channel, which explains the high productivity of OCT-D5A well even though it is a downthrown, downdip well. Based on thin-section description, sandstone percentage exceeds nearly 50%.

Fig. 20 **(**Fig. 20**)** along layer 75 shows strong development in sandstone percentage, width, and channel direction, with reduced sinuosity, resulting in a very wide and continuous channel. The selected wells in this layer are located within the sandstone channel; according to thin-section description, sandstone percentage is nearly 70%. Layer 75 represents the basal section of the K4 zone and exhibits a high productivity index, as most wells produce more from the basal part due to the thick and high-quality sandstone.

## Discussion

### Structural and Stratigraphic Framework of the Nukhul Formation

More precise reconstruction of stratigraphic geometries is now possible thanks to historical datasets gathered by numerous operators between 1980 and 2020 and freshly reprocessed interpretations. The F2 fault dominates the structural configuration, providing first-order control over accommodation, sediment routing, and reservoir compartmentalization (Fig. 12). Juxtaposition relationships show that K4 sandstones are locally juxtaposed with Thebes carbonates or Lower Rudeis shale, resulting in dramatic lithological differences that influence fluid interactions and saturation trends. This is similar with regional structural patterns observed across the Suez rift, where fault-bounded depocenters divide syn-rift reservoirs into distinct compartments^18, 22, 29^. The observed down-dip rise in water saturation in well GS173-2, for example, is due to both structural relief and differential fault sealing.

### Depositional System Evolution and Facies Heterogeneity

According to sedimentological and petrographic evidence, the Nukhul Formation was deposited within an alluvial-fan-dominated system with embedded fluvial channel belts, similar to syn-rift basin-margin systems found elsewhere in the Gulf of Suez and the Red Sea ^37, 39, 40^. The presence of coarse conglomerates, polymictic clasts (such as chert, flint, and carbonate pieces), and mud-clast conglomerates indicates episodic high-energy sediment movement caused by active rift-margin faulting (Figs. 7 and 8). Facies stacking patterns in K3 and K4 indicate a transition from proximal fan distributions to more prolonged fluvial channelization. K3 is distinguished by thin, laterally discontinuous sandstone lenses interspersed with shale and limestone, indicating intermittent channel activity under restricted sand supplies. In contrast, K4 has thicker, more laterally persistent channel bodies with distinct north–south patterns that correspond to the regional structural grain. This transition, from conglomerate-rich lower K4 units to progressively sand-rich upper K4 units, shows improved fluvial organization during times of increased flow or more steady subsidence. In the Nukhul system, lower channel sinuosity and broader channel belts in the basal K4 layers are associated with the largest net-to-gross values, enhanced sorting, and higher reservoir performance.

Overall, the Nukhul Formation’s depositional framework reflects the combined influence of (i) syn-rift structural segmentation, (ii) variable sediment supply from uplifted footwalls, and (iii) autogenic channel avulsion processes, which result in a highly heterogeneous stratigraphic reservoir.

### Integrated Petrophysical Architecture and Reservoir Distribution

Petrophysical analysis of the Nukhul Formation reveals a clear distinction between sealing (K1-K2) and productive (K3-K4) periods (Figs. 5 & 7). K1 and K2 have little porosity, a high shale/carbonate content, and minimal sand input, effectively separate the overlaying and underlying flow units. Their vertical continuity improves hydrocarbon retention in the K3 and K4 reservoirs by limiting vertical leakage, which is consistent with syn-rift stratigraphic configurations where lacustrine shales and carbonates serve as regional seals ^37,39–41^.

K3 has significant reservoir potential but limited development due to thin sandstone units, low lateral connectivity, and structural confinement. Static-model results show significant compartmentalization inside K3, with discrete sand lenses rarely coalescing into laterally extensive flow units.

K4 is the main reservoir interval, with a porosity of around 19%, high net-to-gross values, and well-connected channel belts. Static modelling demonstrates consistent north–south channel continuity, with the proportion of sandstone increasing nearer the basal strata, which corresponds to higher productive wells like OCT-D5A. Maps of porosity, net-to-gross, and shale volume show that reservoir quality improves in the northwestern and southeastern flanks, where channel belts broaden and conglomeratic facies are reduced. Hydrodynamically, reservoir performance in K4 is determined by a combination of depositional geometry, diagenetic alteration, and structural segmentation. The high correlation between modelled channel trends and production behaviour emphasizes the need of including interpreted facies pathways into reservoir characterisation methods.

### Diagenetic Controls on Pore Systems and Reservoir Quality

Thin-section petrography reveals that multistage diagenetic processes considerably alter reservoir quality in the Nukhul Formation, enhancing or occluding porosity within multiple facies types (Figs. 7 & 8). The common framework composition of quartz arenite to sublithic arenite indicates sediment derivation from elevated basement blocks and carbonate platforms, which is compatible with syn-rift provenance patterns ^4,37,39,41,42^.

Pore development is governed by five primary cement types:

- Quartz overgrowths, which reflect early to intermediate burying diagenesis, partially block intergranular pores while maintaining optical continuity.
- Dolomite and dolomicrite, which occur as both replacement textures and pore-filling phases, were formed after quartz overgrowths and feldspar alteration.
- Calcite cement, which forms sparse pore-filling mosaics, is most common in lithic-rich facies.
- A late-stage, pore-destroying phase known as very coarse anhydrite engulfs previous dolomite and quartz overgrowths, forming extensive cement masses indicative of reflux or burial-driven sulphate precipitation.
- Iron oxide coatings, which represent early oxidative conditions, have a local influence on the nucleation of subsequent cement phases.

Secondary porosity is caused by the partial dissolving of dolomite and feldspar, which creates microporosity and increases reservoir capacity locally (Fig. 8). However, intervals with ubiquitous anhydrite and calcite cementation have much lower permeability, especially in conglomeratic facies where compactional forces tighten the pore network.

This diagenetic sequence—quartz overgrowth → dolomite → calcite → anhydrite—is consistent with existing syn-rift diagenetic models and explains the significant vertical and lateral variation seen between wells. The best reservoir quality corresponds to well-sorted K4 channel sandstones with limited late cementation, while poorly sorted conglomerates and lithic-rich beds often have the lowest permeability.

### Implications for Reservoir Connectivity, Compartmentalization, and Field Development

**Fig. 21:**
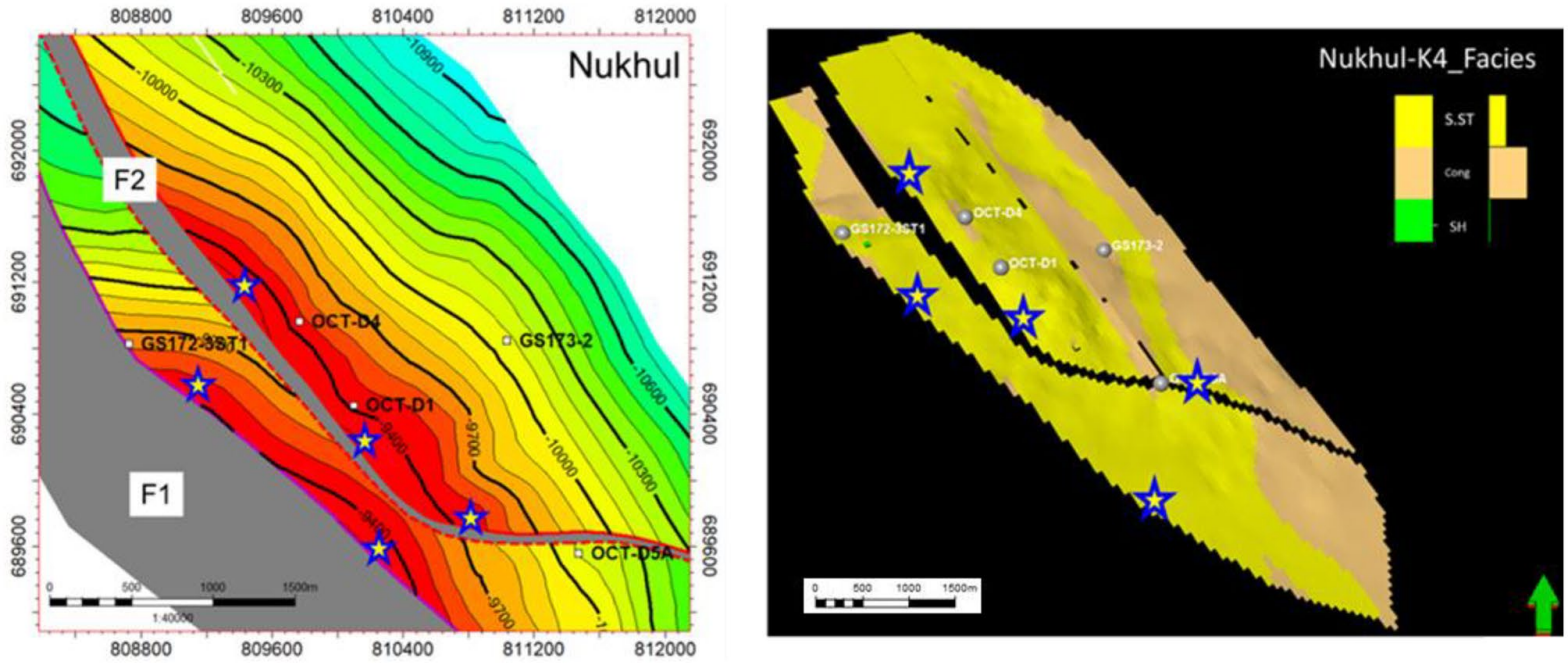
**S**tructure contour map and a static model layer to demonstrate how the model fit to support the concept of proposed opportunities presence (bright stars) through structural and facies depict Nukhul reservoir

Several untested structural and stratigraphic traps have been discovered along footwall culminations, relay ramp crests, and in zones where converging channel belts are locally constrained by structural relief. These areas are interpreted as prospective attic storage places and pay zones that have been skipped, as shown in **(**Fig. 21**)**. Reservoir sweet spots are typically found in structurally elevated areas with higher sandstone maturity, thinner conglomeratic facies, and lower cementation intensity. These zones are associated with lower shale volumes, decreased late-stage anhydrite cement, and increased porosity, creating favourable circumstances for subsequent well placement, as shown in **(**Fig. 21**)**. Fault-seal analysis also indicates that certain faults have sufficient gouge and smear characteristics to operate as partial or complete baffles to cross-fault flow. This is consistent with field-wide pressure variations and varying initial water cut in wells straddling the same structural blocks. Structurally controlled variations in oil–water contact geometry reinforce compartmentalization, including spill points and tilt effects that match observed production behaviour. The combination of seismic facies, structural closure, reservoir-quality mapping, and static modelling reveals numerous undrilled structural culminations capable of supporting new development wells.

The structural architecture of the study region has a first-order impact on reservoir connectivity, fluid dispersion, and compartmentalized production performance. The Nukhul Formation is part of an asymmetric half-graben system, with channelized sandstone bodies and conglomeratic wedges distributed by large listric faults, relay ramps, and secondary antithetic faults. Fault throws locally surpass channel thickness, resulting in reservoir juxtaposition against sealing mudstones and different pressure cells characteristic of Gulf of Suez syn-rift traps^37,39–41^.

Stratigraphic-structural interaction is most visible in the K4 interval, where north–south trending channels connect fault damage zones. Where these channels intersect high-throw faults, reservoir continuity is reduced, and production responses differ significantly between adjacent wells. Whereas throw diminishes throughout relay ramps, channel belts stay laterally connected, enhancing overall productivity.

The developed updated static model will be helpful in choosing the most valid locations characterized by high quality and good reservoir extension, which is beneficial for the development plan, as shown in **(**Fig. 21**)**. Integration of the new static model indicates that fault geometries have greater influence on reservoir continuity than previously thought, as shown in **(**Fig. 21**)**. The refined model includes updated fault sticks, revised stratigraphic layering, and newly generated facies bodies, allowing for more accurate predictions of sandstone connectivity, particularly within K4, where channel belts thicken and achieve greater lateral persistence near rollover crests and footwall highs. Some well opportunity ideas were suggested **(**Fig. 21**)** based on the structural model scenario as an attic location. These were verified with the static model results and clarified with bright stars as locations for new proposed ideas. One of these opportunities, which was close to OCT-D well, was approved as one of the next well opportunities on the rig schedule. The study recommends and gives alerts to take into consideration that there are different proposed areas that need to be developed as shown in **(**Fig. 21**)**.

The different stars allocated on the map indicate that there are hot areas that have not been penetrated, as there are still attic locations; the well spacing between wells is too large, which indicates that the area needs more wells, in addition to facies change enhancement and the direction of reservoir quality improvement. So, it is recommended to drill those proposed stars as an oil acceleration strategy for more oil production and oil reserve. Attic locations will have lower water saturation than current wells; in addition, the new wells will also be oriented toward facies enhancement, which will improve the oil rate and give the reservoir a longer lifespan, increasing the oil reserve. At the same time, the new wells may replace existing wells that have mechanical issues or whose oil rate is lower than expected. All of this will result in significant cost savings because drilling from sidetracks from current wells is far less expensive than drilling from new slots. This means more oil production by increasing the daily oil rate at low cost, along with increased oil reserve. Several of these targets have the potential to improve field production by an estimated 3,000–4,000 BOPD, depending on cumulative reservoir quality, sandstone dispersion, and flow-unit characteristics.

Overall, structural structure, associated with regional fault-seal behaviour, remains an important influence on reservoir connection, remaining oil distribution, and optimal infill well aiming in the Nukhul system.

## Conclusions

An integrated structural-sedimentological-petrophysical approach was applied to the massive October Field, resulting in a significantly revised characterisation of the Nukhul Formation. The key results as the following:

- Updated 3D structural modelling, which was backed by seismic data reinterpretation and the addition of newly acquired well information, offered better limits on fault geometries, fault extents, and main boundary surfaces. This structural framework served as the foundation for developing an updated 3D static model for the Nukhul sandstone reservoir, with a special emphasis on the K4 interval.
- The Nukhul Formation is thought to have an alluvial-fan-dominated depositional system with embedded fluvial channels, similar to those found on syn-rift basin margins. This environment created extremely diverse conglomerate and sandstone assemblages, resulting in significant vertical and lateral facies variability. High-resolution well correlations allowed for the delineation of four zones (K1-K4), with K1-K2 serving as regionally effective seals due to their dominance of low-porosity limestone, shale, and basaltic layers, and K3 consisting of discontinuous, moderately linked sandstone bodies.
- The K4 zone is the primary reservoir interval. It consists of thick, laterally linked channelized sandstones that trend primarily north–south, with sandstone proportion, channel width, and reservoir maturity rising upward. Petrographic and diagenetic investigations show that grain sorting, the distribution of quartz, dolomite, calcite, and anhydrite cements, and localized dissolution processes all influence reservoir quality. These interactions produce heterogeneous permeability while preserving high primary porosity in well-sorted sandstones.
- The structural framework has first-order control over reservoir connectivity and hydrocarbon distribution. The F2 fault is crucial to compartmentalization; juxtaposition relationships and throw size imply effective sealing capacity across key segments, allowing hydrocarbons to be preserved for attic accumulations to form.
- The integrated static model combines structure, facies, porosity, and shale distribution into a geologically consistent framework. The model identifies several undrilled reservoir extensions, high-quality sandstone bands, and probable bypassed pay zones. These traits identify many potential regions for field redevelopment, near-field exploration, and infill drilling. The improved model improves reservoir continuity prediction, clarifies oil–water contact geometry, and increases the ability to identify zones capable of adding reserves and maintaining production.
- Overall, the new geological and structural interpretation improves our understanding of reservoir architecture and provides a solid foundation for optimizing development plans in October Field. The integrated methodology shown here is broadly transferable and can be used in various syn-rift basins throughout the world where alluvial-fan and fluvial depositional systems are heterogeneous and have similar structural complexity.

## Funding declaration

This to confirm this research has not received any funding from any organization.

## Data availability:

Datasets used in this research are available with the corresponding author email: (mostafakhatab@gstd.sci.cu.edu.eg) upon reasonable request.
